# Fusion Toxin BLyS-Gelonin Inhibits Growth of Malignant Human B Cell Lines In Vitro and In Vivo

**DOI:** 10.1371/journal.pone.0047361

**Published:** 2012-10-09

**Authors:** Troy A. Luster, Ipsita Mukherjee, Jeffrey A. Carrell, Yun Hee Cho, Jeffrey Gill, Lizbeth Kelly, Andy Garcia, Christopher Ward, Luke Oh, Stephen J. Ullrich, Thi-Sau Migone, Robin Humphreys

**Affiliations:** 1 Department of Oncology Research, Human Genome Sciences, Inc., Rockville, Maryland, United States of America; 2 Department of Lead Development, Human Genome Sciences, Inc., Rockville, Maryland, United States of America; 3 Department of Immunology Research, Human Genome Sciences, Inc., Rockville, Maryland, United States of America; University of Navarra, Center for Applied Medical Research, Spain

## Abstract

B lymphocyte stimulator (BLyS) is a member of the TNF superfamily of cytokines. The biological activity of BLyS is mediated by three cell surface receptors: BR3/BAFF-R, TACI and BCMA. The expression of these receptors is highly restricted to B cells, both normal and malignant. A BLyS-gelonin fusion toxin (BLyS-gel) was generated consisting of the recombinant plant-derived toxin gelonin fused to the N-terminus of BLyS and tested against a large and diverse panel of B-NHL cell lines. Interestingly, B-NHL subtypes mantle cell lymphoma (MCL), diffuse large B cell lymphoma (DLBCL) and B cell precursor-acute lymphocytic leukemia (BCP-ALL) were preferentially sensitive to BLyS-gel mediated cytotoxicity, with low picomolar EC_50_ values. BLyS receptor expression did not guarantee sensitivity to BLyS-gel, even though the construct was internalized by both sensitive and resistant cells. Resistance to BLyS-gel could be overcome by treatment with the endosomotropic drug chloroquine, suggesting BLyS-gel may become trapped within endosomal/lysosomal compartments in resistant cells. BLyS-gel induced cell death was caspase-independent and shown to be at least partially mediated by the “ribotoxic stress response.” This response involves activation of p38 MAPK and JNK/SAPK, and BLyS-gel mediated cytotoxicity was inhibited by the p38/JNK inhibitor SB203580. Finally, BLyS-gel treatment was shown to localize to sites of disease, rapidly reduce tumor burden, and significantly prolong survival in xenograft mouse models of disseminated BCP-ALL, DLBCL, and MCL. Together, these findings suggest BLyS has significant potential as a targeting ligand for the delivery of cytotoxic “payloads” to malignant B cells.

## Introduction

B lymphocyte stimulator (BLyS), also known as B cell activating factor belonging to the TNF family (BAFF), is a member of the TNF superfamily of cytokines. BLyS is produced by monocytes, macrophages, neutrophils, dendritic cells, and bone marrow stromal cells [1], [2], [3], and is known to be critical for the maintenance of normal B cell development and homeostasis [4]. Full-length BLyS is a type II transmembrane protein with a carboxy terminal extracellular domain, and like most other TNF ligands, is cleaved to release a soluble form [1]. The biological effects of BLyS are mediated by three receptors known as BLyS receptor 3 (BR3, also known as BAFF-receptor (BAFF-R)), transmembrane activator and CAML interactor (TACI), and B cell maturation antigen (BCMA). BLyS binds BR3 and TACI with higher affinity than BCMA, suggesting the BLyS/BCMA interaction *in vivo* may be of less significance [4], [5], [6]. In this regard, the BLyS homolog a proliferation inducing ligand (APRIL) binds BCMA with higher affinity than BLyS and is thought to be the more biologically active ligand for this receptor [5], [6], [7]. All three receptors are expressed almost exclusively among B cell lineages, although the pattern of expression depends upon the stage of B cell development. For example, BCMA is expressed primarily on terminally differentiated mature plasma cells, while BR3 and TACI are expressed on less differentiated B cells [8].

BLyS receptors are also expressed on a broad range of B cell non-Hodgkin lymphomas (NHLs), including mantle cell lymphoma (MCL), diffuse large B cell lymphoma (DLBCL), Burkitt's lymphoma (BL), follicular B cell lymphoma (FL), chronic lymphocytic leukemia (B-CLL), B cell precursor acute lymphocytic leukemia (BCP-ALL), and multiple myeloma (MM) [9], [10], [11], [12], [13], [14], [15]. B cell NHLs are a heterogeneous group of lymphoid cancers with differing patterns of clinical behavior and responses to therapy [16]. Most NHLs respond to initial treatment, but ultimately recur as chemoresistant disease. Although the addition of rituximab to therapeutic regimens has generally improved clinical outcomes, new therapeutic agents are needed.

The use of antibodies or ligands to deliver toxins to specific receptors on targets cells has received significant attention over the past decade [17]. In 1999, the FDA approved the use of an IL-2-diphtheria toxin fusion protein (denileukin difitox) for treatment of cutaneous T cell lymphoma. More recently, an immunotoxin targeting *Pseudomonas* exotoxin to CD22 on B cells (BL22) has generated exciting results in clinical trials of hairy cell leukemia [18], [19], [20]. Similar immunotoxins targeting cells expressing CD19 and CD25 are currently being tested in humans as well [17]. Chimeric proteins composed of chemokine ligands, such as IL-3, IL-13, GM-CSF and VEGF, fused to various toxins have also been generated [17], [21], [22], [23]. These drugs have shown specific cytotoxicity against target cells, efficacy in animal models of cancer, and several are currently under clinical investigation.

Based on the B cell restricted expression of BLyS receptors, Nardelli et. al. suggested that BLyS has significant potential as a targeting agent for B cell NHLs [24]. As proof of concept, radiolabeled BLyS was shown to specifically and rapidly localize to B cell tumors in mice [25] and in humans [26]. More recently, BLyS has been used to deliver the plant toxin gelonin to B cells [27], [28], [29], [30]. Gelonin is a type I ribosome inactivating protein (RIP) originally isolated from seeds of the *Gelonium multiflorum* plant [31]. RIPs are *N*-glycosidases that remove a specific adenine from the highly conserved α-sarcin/ricin loop of eukaryotic 28S rRNA [32]. This inactivates ribosomes and inhibits protein synthesis leading to cell death. Importantly, unlike type II RIPs, type I RIPs lack the lectin-like B chain required to bind and enter cells on their own. Thus, gelonin lacks toxicity unless conjugated or fused to a molecule that can be internalized by target cells. Rosenblum and colleagues have demonstrated that a recombinant BLyS-gelonin fusion toxin (rGel/BLyS) is highly cytotoxic against malignant NHL cell lines, especially MCLs and DLBCLs [27], [28]. The fusion toxin was internalized by target cells and the cytotoxic effects could be blocked by soluble BLyS receptors. In a separate study, Nimmanapalli et. al. showed that rGel/BLyS bound to BR3^+^/CD19^+^ cells from B-CLL patients and induced annexin V binding [29], suggesting the drug induces apoptosis of primary B-CLL cells. Here, using a similar BLyS-gelonin fusion toxin (BLyS-gel), these findings are expanded using a larger and more diverse panel of B cell NHL cell lines and xenograft models of BCP-ALL, DLBCL, and MCL. The results provide additional *in vitro* and *in vivo* evidence that BLyS-mediated delivery of cytotoxic agents may be an effective strategy for the treatment of B cell malignancies.

## Materials and Methods

### Ethics Statement

All mouse studies were conducted in strict accordance with the recommendations in the Guide for the Care and Use of Laboratory Animals of the National Institutes of Health. The study protocol was approved by the IACUC of The American Red Cross (OLAW assurance number A3379) and all efforts were made to minimize suffering.

### Cell lines and culture conditions

All cell lines were obtained from the American Type Culture Collection (ATCC, Manassas, VA) or the German Collection of Microorganisms and Cell Cultures (DSMZ, Braunschweig, Germany). All B cell lines were cultured in RPMI+10% FBS (Invitrogen, San Deigo, CA) at 37°C in a humidified atmosphere with 5% CO_2_. All other cell lines were cultured under the same atmospheric conditions in media recommended by the supplier.

### Flow cytometry

Cells in suspension were pelleted, washed in PBS, and resuspended in FACS buffer (PBS with 0.1% BSA and 0.1% sodium azide). Adherent cells were detached from culture flasks using Enzyme-Free Cell Dissociation Buffer (Invitrogen). Cell surface expression of TACI and BCMA was determined using biotinylated receptor-specific goat antibodies (R&D Systems Inc., Minneapolis, MN) detected with PE-conjugated streptavidin (Dako, Carpinteria, CA). BR3 surface expression was determined using a BR3-specific AlexaFluor647-conjugated mouse antibody (Axxora, San Deigo, CA). Non-specific, species isotype matched antibodies were used as negative controls (R&D Systems or BD Biosciences, San Jose, CA). Cells were incubated with antibodies for 20 minutes at room temperature, washed, resuspended in FACS buffer containing 0.5 µg/ml propidium iodide, and analyzed on a FACSCalibur instrument (BD Biosciences).

### Viability and caspase activity assays

Cells were seeded in opaque white 96-well polystyrene plates (Corning, Acton, MA) at a density of 5-10×10^3^ cells/well in 50 µl of culture media. An additional 50 µl of media was added containing various concentrations of BLyS-gel (see supplementary information for BLyS-gel construction and production details) or free gelonin (Axxora) treatments. TACI-Fc and a control Fc-fusion (cntl-Fc) composed of the extracellular domain of human Robo4 fused to the Fc region of human IgG1 were prepared in-house and used at 5 µM as BLyS blocking reagents. For experiments using BR3, TACI, or BCMA receptor blocking antibodies (R&D Systems), treatments were prepared with blocking antibodies at 2 µg/ml. In other studies the TRAIL-R1 agonistic antibody mapatumumab (Human Genome Sciences, Inc, Rockville, MD) was used at 10 µg/ml, general caspase inhibitor z-VAD-FMK (BD Biosciences) was used at 10 µM, the lysosomotropic drug chloroquine (Sigma-Aldrich, St. Louis, MO) was used at 100 µM, and the p38/JNK inhibitor SB203580 (Promega, Madison, WI) was used at various at concentrations up to 12.5 µM. Cells were incubated at 37°C for 72 hrs and viability was measured by adding 50 µl of the Cell Titer-Glo or Caspase-Glo 3/7 reagent (Promega) directly to cells in culture media. The cells were agitated for 5 min at room temperature and the luminescent signal was read using a Wallac Envision 2100 plate reader (Perkin-Elmer, Boston, MA). All treatments were performed in triplicate and each experiment was performed at least twice. The average and standard deviation were determined and plotted using Prism software (GraphPad, San Diego, CA). Viability data are presented relative to the viability of untreated cells, which was arbitrarily set to one.

### Internalization assay

Cells were pelleted, washed in PBS, resuspended in FACS buffer, and incubated with 100 nM BLyS-gel or gelonin for 2 hrs at room temperature. Surface bound proteins were stripped by washing cells in glycine buffer (500 mM NaCl, 0.1 M glycine, pH 2.5) for 5 min followed by neutralization in 0.5 M Tris (pH 7.4). Cells were then fixed in 4% paraformaldehyde for 30 min and permeablized with 0.2% Triton-X100 in PBS for 10 min. Finally, cells with internalized BLyS-gel or gelonin were detected using a gelonin-specific antibody (see supplementary information) in 0.1% Tween-20 by flow cytometry.

### Protein synthesis assay

Protein synthesis was analyzed by measuring incorporation of Click-iT HPG (homopropargylglycine, an L-methionine analog) using the Click-iT Cell Reaction Buffer kit (Invitrogen). Cells (0.3×10^6^) were seeded into wells of 6-well plate and treated with BLyS-gel at 500 pM for 0, 24, 48, or 72 hrs. Cells were then washed, resuspended in L-methionine free media, and incubated for 30 min to deplete methionine reserves. Next, Click-iT HPG was added at a final concentration of 50 µM and cells were incubated another 4 to 16 hrs. As a positive control for protein synthesis inhibition, cells were treated with cycloheximide (Sigma-Aldrich) at 1 µg/ml for 4 hrs prior to addition of Click-iT HPG. To detect Click-iT HPG incorporation cells were washed in PBS+0.5% BSA, fixed in 4% paraformaldehyde for 10 min, permeablized briefly in 0.25% Triton X100 in PBS, and incubated for 30 min with Click-iT reaction cocktail including AlexaFluor647 azide. Lastly, cells were washed in PBS with 0.5% BSA and counterstained with propidium iodide for analysis by flow cytometry.

### Preparation of cell lysates

Cells were cultured in 6-well plates and treated with BLyS-gel or BLyS at 500 pM for 0, 4, 8, 24, 48 or 72 hrs. For some experiments treatments were combined with the p38/JNK inhibitor SB203580 at 12.5 µM. As a positive control for induction of the ribotoxic stress response, cells were treated with 1 µg/ml anisomycin (Sigma-Aldrich) for 30 min. At the end of the treatment period cells were washed in PBS and lysed in ice-cold RIPA buffer (25 mM Tris-HCl, 150 mM NaCl, 1% Triton X-100, 0.5% sodium deoxycholate, 0.1% SDS) supplemented with protease and phosphatase inhibitor tablets (Roche, Indianapolis, IN). Protein concentrations were determined using a modified Bradford Assay (Coomassie Plus; Pierce, Rockford, IL).

### Western blot analysis

Proteins were separated on 4–12% NuPAGE Bis-Tris gels (Invitrogen) and transferred to Invitrolon PVDF membranes (Invitrogen) for western blot analysis. Membranes were blocked with a solution of 5% milk (w/v) in TBS/T or 5% BSA (w/v) in TBS/T for phospho-specific antibodies. The following primary antibodies were used: phospho-p38 MAPK (pT180-pY182), p38 MAPK, phospho-JNK/SAPK (pT183-pY185), JNK/SAPK, caspase-9, PARP, cleaved PARP, β-tubulin and α-tubulin (Cell Signaling Technology, Beverly, MA). Membranes were probed with antibodies diluted 1∶500 to 1∶5,000 in blocking buffer overnight at 4°C, washed 3× in TBS/T, and detected using HRP-conjugated secondary antibodies (Cell Signaling) diluted 1∶10,000 and enhanced chemiluminescence substrate (Invitrogen).

### Quantitation of p38 phosphorylation

Rec-1 cells were seeded in 48-well plates at a density of 5×10^5^ cells/well in 500 µl of culture media and treated with BLyS-gel at 500 pM for 24 hrs or anisomycin at 1 µg/ml for 1 hr. At the end of the treatment period cell lysates were prepared as described above. Ten microliters of cell lysates were transferred to wells of an opaque white 96-well ½ area polystyrene plates (Corning, Acton, MA) for quantitation of p38 phosphorylation using the AlphaScreen SureFire p38 MAPK (p-Thr180/Tyr182) assay kit (Perkin-Elmer) according to manufacturer's protocol. The luminescent signal was read using an Envision 2104 plate reader (Perkin-Elmer). Results are presented relative to the signal obtained from untreated cells, which was arbitrarily set to one.

### Xenograft models of disseminated B-NHL

Nalm-6 (BCP-ALL) or Rec-1 (MCL) cells in log-phase growth (1×10^6^) were injected into the tail veins of 6–8 week old female SCID mice on day 0. Likewise, NUDHL-1 cells were injected into female NOD.SCID/IL2Rγ null mice (NSG; The Jackson Lab, Bar Harbor, ME). Mice were then divided into groups (n = 10) for treatment with vehicle, free gelonin, or BLyS-gel. On day 1, all mice were injected i.v. with 5 mg/kg of the murine BLyS-specific antibody 10F4 (prepared in-house) to deplete circulating murine BLyS. On day 2, treatments were initiated using the dose and schedule indicated in the figure legends. Additional 10F4 was given prior to each new week of treatment. Mice injected with Nalm-6 or Rec-1 cells were monitored twice a week until body weight loss equaled 20% of starting weight or signs of hind limb paralysis were observed, at which point they were sacrificed. Mice injected with NUDHL-1 cells developed multiple signs of disease; therefore, these mice were sacrificed when i) the largest externally palpable tumor was 20 mm in diameter, ii) hind limb paralysis developed, or iii) eyes became too enlarged to close. Survival data are plotted as Kaplan-Myer survival curves and differences were analyzed for significance using the Logrank test.

### Analysis of BLyS-gel localization to Rec-1 cells *in vivo*

SCID mice were injected i.v. with Rec-1 cells as described above. When mice began to lose weight they were injected i.v. with 2 mg/kg gelonin or BLyS-gel. Mice were euthanized 4 or 24 hrs later for collection of spleens or bone marrow, respectively. The mean fluorescence intensity of hCD19^+^ Rec-1 cells in bone marrow aspirates or homogenized spleens that were stained using the anti-gelonin pAb was determined by flow cytometry.

### Analysis of BLyS-gel treatment on tumor burden in spleens of mice with “established” disease

SCID mice were injected i.v. with Rec-1 cells as described above. The presence of circulating human β2-microglobulin (hβ2M) in mouse serum was used to monitor disease progression [33]. Blood was collected on day 25 from the retro-orbital sinus and serum was analyzed for the presence of hβ2M using a quantitative sandwich ELISA kit (Alpha Diagnostics, San Antonio, TX). Serum from a naïve mouse was used a negative control. Mice with detectable levels of hβ2M in the serum were injected i.v. with 2 mg/kg gelonin or BLyS-gel. Seventy-two or 120 hrs later, mice were sacrificed and spleens were harvested. Fixed-formalin paraffin-embedded (FFPE) tissue blocks were prepared and sections were stained with a human CD20-specific antibody (Diagnostic BioSystems, Pleasanton, CA) to detect the Rec-1 cells.

## Results

### Construction and characterization of BLyS-gel

The BLyS-gel fusion toxin was designed such that gelonin was fused to the NH_2_-terminus of BLyS. This arrangement was chosen because structural studies indicate the COOH-terminus of natural BLyS is critical for receptor binding [34]. SDS-PAGE analysis of purified BLyS-gel under non-reducing and reducing conditions identified bands of approximately 45 kD (Fig. S1A), which is the predicted size of BLyS-gel monomers. Western blot analysis using BLyS- or gelonin-specific antibodies also identified this band (Fig. S1A), confirming the presence of both components in the fusion toxin. Importantly, fusion of gelonin to BLyS did not affect the affinity of BLyS for its receptors (Table S1).

The active BLyS molecule is a non-covalently linked homotrimer [35]. To verify BLyS-gel was active and retained the ability to bind B cells expressing BLyS receptors, several malignant B cell lines were incubated with BLyS-gel or free gelonin and binding was analyzed by flow cytometry (Fig. S1B). BLyS-gel bound to all B cell lines tested, but free gelonin did not, indicating binding was mediated by the BLyS moiety of the BLyS-gel molecule. Neither BLyS-gel nor free gelonin bound to Jurkat T cells (Fig. S1B), which lack BLyS receptors [10], [36], [37]. In addition, BLyS-gel binding to SUDHL-4 cells was competed by recombinant human BLyS (Fig. S1C), providing further evidence that the BLyS component of BLyS-gel is active and responsible for the ability to bind BLyS receptors on B cells.

### BLyS-gel treatment reduces the viability of specific subtypes of malignant B cell lines

**Figure 1:**
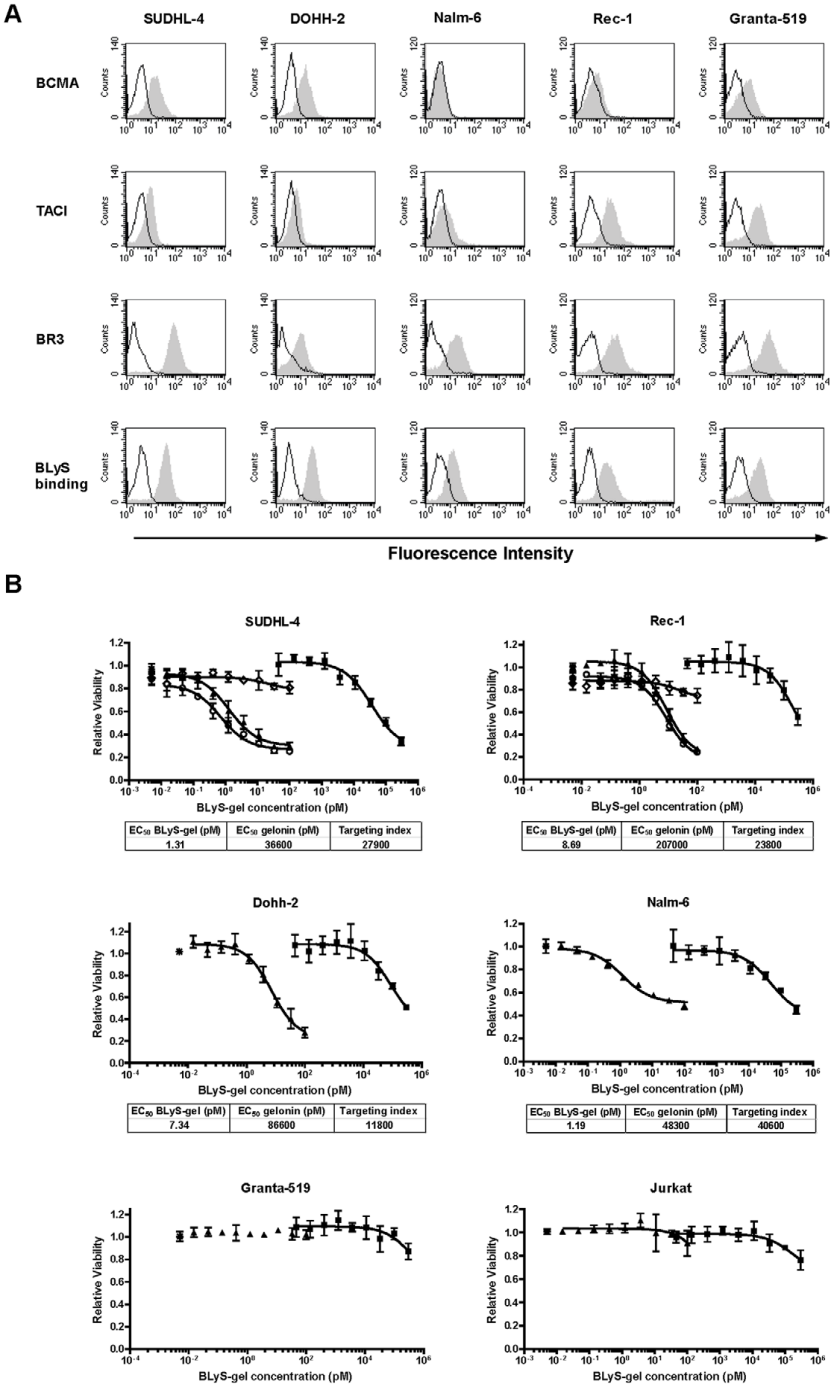
BLyS receptor expression and sensitivity to BLyS-gel in selected malignant B cell lines. **A.** BLyS receptor expression was determined by flow cytometry. The indicated cell lines were stained with antibodies specific for the BLyS receptors BCMA, TACI or BR3. Alternatively, the ability of cells to bind BLyS was determined by incubation with biotinylated BLyS. Black line, detection reagents only; gray-shaded peak, BLyS receptor antibodies or BLyS-biotin plus detection reagents. **B.** Cells were treated with a serial dilution of gelonin (▪; dashed line) or BLyS-gel (▴) in the absence or presence of TACI-Fc (◊) or cntl-Fc (○) at a fixed concentration of 5 µM. Cells were also treated with TACI-Fc (♦) or cntl-Fc (•) alone at 5 µM for comparison. Cell viability was analyzed following 72 hrs of treatment. Data are presented as viability relative to cells treated with media only (*). EC_50_ values for sensitive cells lines are shown below the graphs. Targeting index = EC_50_ gelonin/EC_50_ BLyS-gel

A panel of malignant B cell lines was screened for cell surface expression of BLyS receptors by flow cytometry. The cells were also screened for the ability to bind BLyS, which was used as a surrogate to predict binding of BLyS-gel. All of the cell lines in the panel expressed at least one BLyS receptor and were able to bind BLyS (Fig. 1A). BLyS-gel treatment for 72 hrs substantially reduced the viability of 4/5 of these B cell lines, with EC_50_ values in the low picomolar range (Fig. 1B). Free gelonin reduced viability of the same four cell lines, but at much higher concentrations, resulting in targeting indices greater than 10,000-fold (Fig. 1B). Jurkat T cells, which do not express BLyS receptors, were not sensitive to BLyS-gel or free gelonin. To demonstrate the effect was BLyS-mediated, SUDHL-4 and Rec-1 cells were also incubated with the extracellular domain of the BLyS receptor TACI fused to the Fc region of human IgG1 or a control Fc-fusion. As expected, TACI-Fc blocked the cytotoxicity of BLyS-gel while the control Fc-fusion did not (Fig. 1B).

Next, a much larger panel of malignant B cell lines was screened for cell surface expression of BLyS receptors and sensitivity to BLyS-gel (Table 1). No obvious patterns emerged correlating BLyS receptor expression to BLyS-gel sensitivity. However, several subtypes of B cell malignancies were preferentially sensitive to BLyS-gel treatment. Specifically, 5/5 BCP-ALL cell lines, 5/6 MCL cell lines, and 5/12 DLBCL cell lines were at least partially sensitive to BLyS-gel. The DLBCL cell line SUDHL-8 may be mischaracterized and is not expected to be sensitive to BLyS-gel since it lacks cell surface BLyS receptors. Likewise, BLyS-gel was not cytotoxic to any other cell line lacking expression of BLyS receptors, indicating BLyS-gel cytotoxicity is BLyS receptor mediated. Importantly, a number of insensitive cell lines express BLyS receptors, suggesting BLyS receptor expression is necessary, but not always sufficient for sensitivity to BLyS-gel.

**Table 1:** Cell surface expression of BCMA, TACI, BR3 and comparative sensitivity of various cancer cell lines to BLyS-gel.

Cell line	Cell type	BCMA*	TACI*	BR3*	BLyS-gel sensitivity**
Granta-519	MCL	4.2	14.5	41.1	-
Jeko-1	MCL	0.0	7.0	58.0	+++
JVM-2	MCL	31.5	76.3	14.9	+
Mino	MCL	2.7	13.2	0.7	++++
Rec-1	MCL	1.8	20.7	29.9	+++
Z138	MCL	3.5	10.7	51.8	+++
DB	DLBCL	2.8	0.9	170.9	+++
HT	DLBCL	0.2	−0.8	90.7	-
Karpas-422	DLBCL	0.4	1.7	74.4	-
NUDHL-1	DLBCL	−0.2	42.0	54.1	++++
Pfeiffer	DLBCL	41.3	−0.1	−0.1	-
RL	DLBCL	2.8	−0.2	20.3	-
SUDHL-4	DLBCL	10.1	4.4	74.8	+++
SUDHL-5	DLBCL	1.0	−0.2	20.6	+++
SUDHL-6	DLBCL	13.7	19.7	57.2	-
SUDHL-8	DLBCL(?)	−0.1	0.2	0.1	-
Toledo	DLBCL	1.6	42.1	2.8	++
WSU-DLCL2	DLBCL	6.8	0.7	48.5	-
BJAB	BL	2.0	0.0	6.0	-
Namalwa	BL	16.1	16.0	7.6	-
Raji	BL	−0.5	0.6	3.2	-
Ramos	BL	4.3	−0.3	10.4	-
ST486	BL	10.6	8.5	−0.2	-
KMS-12-BM	MM	14.8	−1.2	6.1	-
KMS-12-PE	MM	12.1	−0.1	0.3	-
MC/CAR	MM(?)	6.0	57.9	38.1	-
NCI-H929	MM	11.7	−0.5	−0.2	-
RPMI-8226	MM	9.7	15.4	0.0	-
U266	MM	4.9	−0.7	0.1	-
CCRF-SB	BCP-ALL	3.4	30.1	4.4	+
Nalm-6	BCP-ALL	−0.2	1.6	11.8	++
Reh	BCP-ALL	−0.9	0.0	12.1	+
RS4; 11	BCP-ALL	−0.6	−0.3	11.8	+
SUP-B15	BCP-ALL	ND	ND	ND	+
EHEB	B-CLL	7.0	2.0	0.9	-
JVM-3	B-CLL	33.7	29.3	39.4	-
JVM-13	B-CLL	5.6	14.8	36.2	-
MEC-1	B-CLL	1.0	5.9	27.5	-
MEC-2	B-CLL	10.1	23.8	15.9	-
Dohh-2	follicular lymphoma	9.7	2.0	5.2	+++
ARH-77	B lymphoblast	1.2	7.2	9.0	-
IM-9	B lymphoblast	0.1	43.2	4.3	-
JM-1	B lymphoblast	10.3	52.3	23.3	+++
MC-116	B lymphoblast	0.7	−0.4	27.3	++++
SKW 6.4	B lymphocyte	3.8	48.2	38.5	-
HL-60	acute promyelocytic leukemia	−1.4	−1.3	0.1	-
Jurkat	T cell leukemia	ND	ND	ND	-
AsPC-1	pancreatic adenocarcinoma	ND	ND	ND	-
CFPAC-1	pancreatic adenocarcinoma	−0.3	−0.2	3.2	-
A549	lung adenocarcinoma	−1.5	−1.4	1.8	-
HT-29	colon adenocarcinoma	ND	ND	ND	-
LNCaP	prostate adenocarcinoma	ND	ND	ND	-
T98G	neuroblastoma	0.3	0.0	3.2	-

* Values determined by flow cytometry and presented as (MFI 1° Ab+MFI 2°Ab) – (MFI 2° Ab only)

** sensitivity determined by incubation with 500 pM BLyS-gel for 72 hrs. Cell viability determined as described in Materials and Methods. Viability reduced by >20% = +; >40% = ++; >60% = +++; >80% = ++++

(?) cell line possibly mis-characterized

Abbreviations: MFI, mean fluorescense intensity; MCL, mantle cell lymphoma; DLBCL; diffuse large B cell lymphoma; BL, Burkitt's lymphoma; MM, multiple myeloma; BCP-ALL, B cell precursor-acute lymphoblastic leukemia; B-CLL, B cell chronic lymphocytic leukemia; ND, not determined

### BLyS-gel cytotoxicity is mediated primarily by BLyS receptors BR3 and TACI

**Figure 2:**
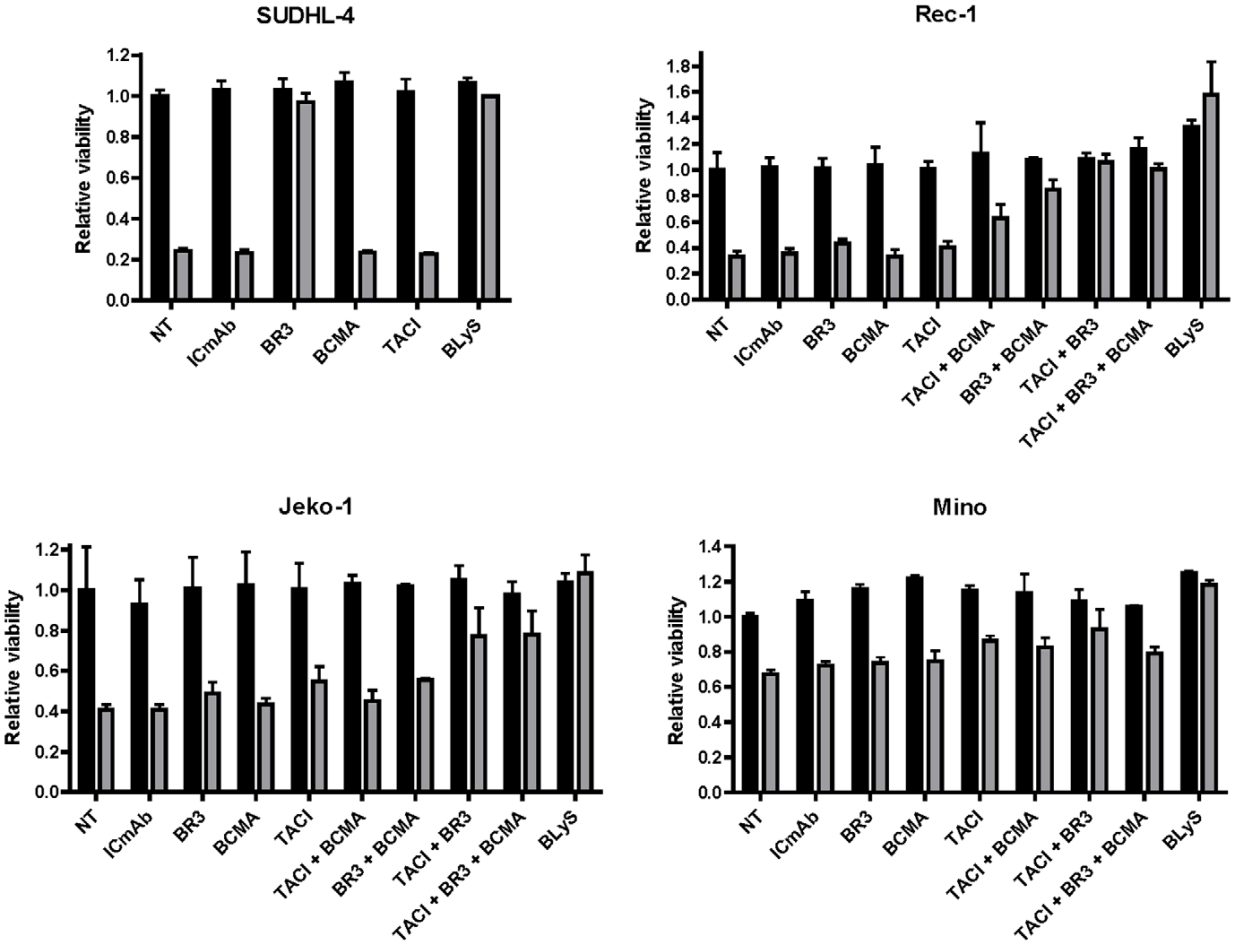
Identification of BLyS receptors responsible for mediating the cytotoxic effects of BLyS-gel. The indicated cell lines were treated with (gray bars) or without (black bars) BLyS-gel at 500 pM. BLyS-gel binding to individual BLyS receptors was blocked using antibodies specific for BR3, BCMA or TACI at 2 µg/ml. A 10-fold excess of recombinant human BLyS was used as a positive control. A non-specific control antibody (ICmAb) was used as a negative control. Cell viability was analyzed following 72 hrs of treatment. NT, no treatment.

Although the data presented in Table 1 indicated that BLyS-gel cytotoxicity was BLyS receptor mediated, the identity of the individual BLyS receptor(s) involved was unclear. Therefore, antibodies able to block BLyS binding to BR3, TACI or BCMA were used to determine which BLyS receptor(s) mediates the cytotoxic effects of BLyS-gel in four sensitive cell lines. The blocking ability of these antibodies was verified by flow cytometry using murine cells that lack endogenous BLyS receptors, but have been stably transfected with expression vectors for human BR3, TACI or BCMA (data not shown). The BR3 or TACI blocking antibodies, either alone or in combination, maximally inhibited BLyS-gel cytotoxicity in the four cell lines tested (Fig. 2). The BCMA antibody contributed a blocking effect only when used in combination with the BR3 or TACI antibodies and only in the Rec-1 cells, despite equivalent or higher BCMA expression in the Mino and SUDHL-4 cells. BLyS completely blocked the cytotoxic effect of BLyS-gel in all four cell lines, which was expected given that BLyS was shown to compete for binding of BLyS-gel (Fig. S1C). Taken together, these data suggest BLyS-gel cytotoxicity is mediated primarily by BR3 and TACI, although the residual BLyS-gel cytotoxicity that is not blocked by the BLyS receptor antibodies in the Jeko-1 and Mino cell lines suggests additional unidentified BLyS receptors may be present on these cells.

### BLyS-gel inhibits protein synthesis in sensitive, but not insensitive, cell lines

Gelonin is an N-glycosidase that inactivates ribosomes and inhibits protein synthesis [31], [32]. To determine whether BLyS-gel treatment inhibits protein synthesis, cells were incubated with BLyS-gel for various periods of time and nascent peptide synthesis was monitored by incorporation of a labeled methionine analog (Table 2). In untreated cells, 70-95% of cells were labeled following the incorporation period. Treatment with the positive control protein synthesis inhibitor cycloheximide reduced the number of labeled cells to less than 1%. BLyS-gel treatment of sensitive cell lines inhibited protein synthesis in a time-dependent manner over a 72 hr period. Treatment of the BLyS-gel insensitive cell line Granta-519 had no effect on protein synthesis.

**Table 2:** BLyS-gel treatment inhibits protein synthesis in sensitive cell lines.

Treatment conditions			HPG incorporation (% positive cells) *			
HPG	CHX	BLyS-gel	SUDHL-4†	Mino†	Jeko-1†	Granta-519‡
-	-	-	0.0	0.0	0.1	0.0
+	-	-	93.1	94.0	71.6	84.7
+	-	24 h	63.2	75.5	38.6	82.1
+	-	48 h	50.9	62.2	29.3	82.4
+	-	72 h	8.7	54.5	33.4	80.1
+	4 h	-	0.1	0.7	0.2	0.1

* values determined by flow cytometry as described in Materials and Methods

† BLyS-gel sensitive

‡ BLyS-gel insensitive

Abbreviations: HPG, homopropargylglycine (analog of L-methionine); CHX, cycloheximide

### BLyS-gel is internalized by sensitive and insensitive cell lines

**Figure 3:**
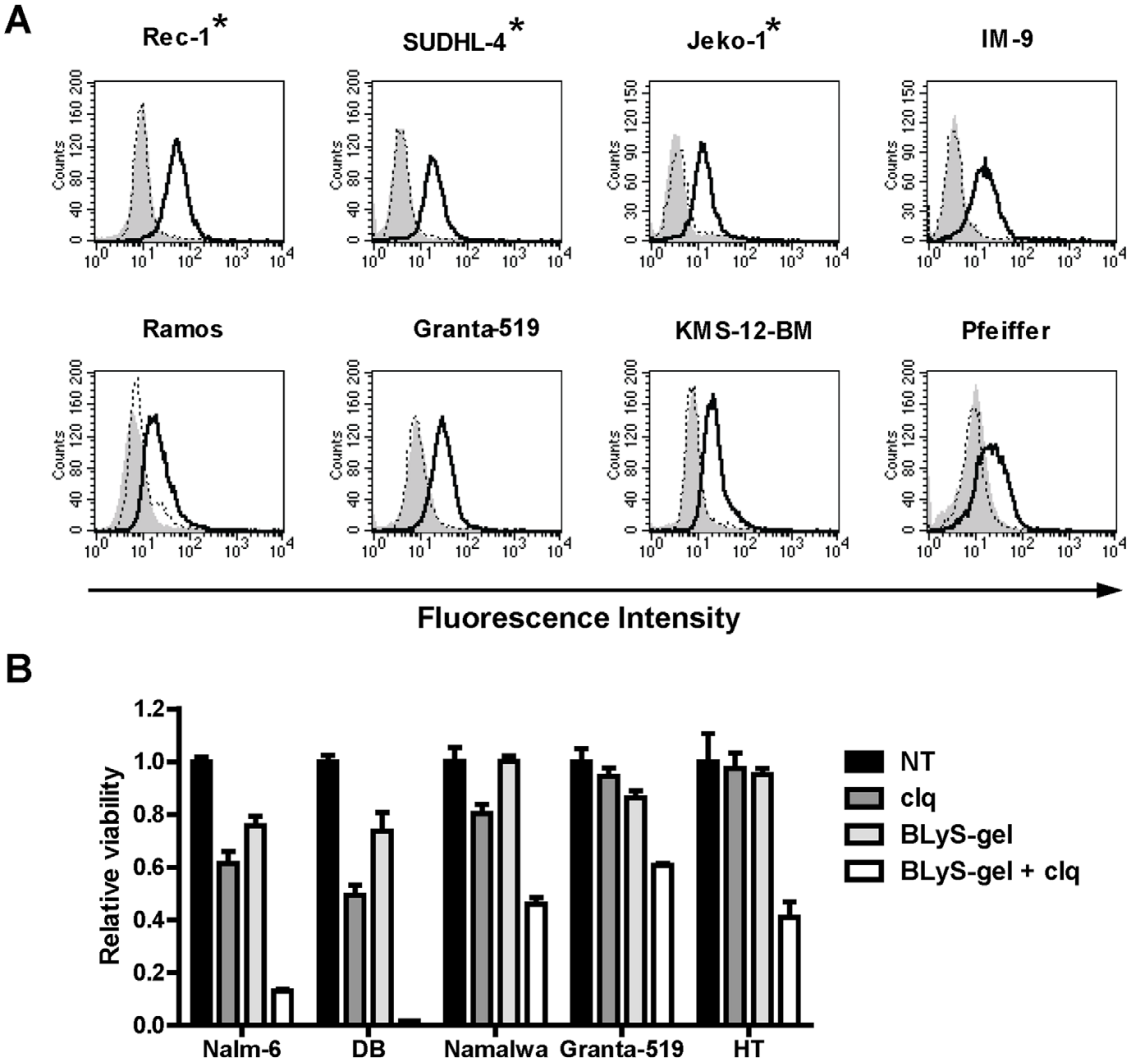
BLyS-gel is internalized and cytotoxicity is enhanced with chloroquine. **A.** BLyS-gel internalization into target cells was analyzed by flow cytometry. Both BLyS-gel sensitive (*) and insensitive lines were tested. Cells were incubated with BLyS-gel or gelonin for 2 hrs. Surface bound proteins were acid-stripped by washing cells in glycine buffer then fixed and permeablized for detection of internalized BLyS-gel or gelonin using a gelonin-specific antibody. Gray-shaded peak, gelonin detection reagents only; dashed line, gelonin plus detection reagents; solid line, BLyS-gel plus detection reagents. **B.** Cells were treated with BLyS-gel at 500 pM for 16 hrs. Chloroquine (clq) was then added at 100 µM for 8 hrs. Cells were washed, resuspended in fresh media, and incubated for an additional 72 hrs. NT, no treatment.

Gelonin must be internalized and enter the cytoplasm of target cells to disrupt ribosome function, inhibit protein synthesis, and cause cytotoxicity. To test the hypothesis that BLyS-gel insensitive cells do not effectively internalize BLyS-gel upon BLyS receptor binding, the cellular uptake of BLyS-gel was analyzed by flow cytometry. Assay conditions were optimized such that cell surface bound BLyS-gel was completely removed by acid-stripping, ensuring that only internalized BLyS-gel was detected with this assay (data not shown). As expected, BLyS-gel, but not free gelonin, was internalized by BLyS-gel sensitive cell lines (Fig. 3A). Interestingly, BLyS-gel was also internalized by insensitive cell lines. These data are consistent with previous findings [27] and indicate that resistance to BLyS-gel by cell lines expressing BLyS receptors is not due to a defect of internalization.

### Chloroquine enhances sensitivity to BLyS-gel

Recent studies indicate that cellular resistance to immunotoxins is due primarily to inefficient release from endosomal vesicles following internalization [38], [39]. Chloroquine is an endosomotropic drug that accumulates in acidic compartments such as late endosomes and lysosomes leading to osmotic rupture of the vesicles, and has been reported to enhance the cytotoxicity of immunotoxins [40]. To determine whether endosomal sequestration was responsible for resistance to BLyS-gel, cells were exposed to BLyS-gel then treated with chloroquine. Chloroquine treatment enhanced the cytotoxic effects of BLyS-gel on all five cells lines tested (Fig. 3B), suggesting BLyS-gel is not effectively released from endosomes in resistant cells.

### BLyS-gel induced cell death is caspase-independent

**Figure 4:**
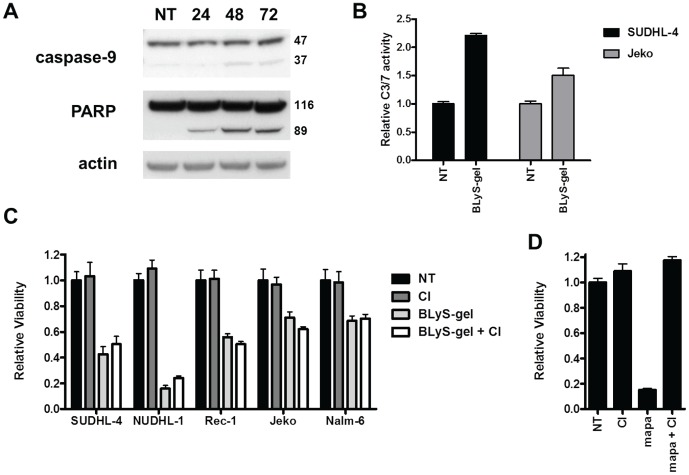
BLyS-gel mediated cytotoxicity is caspase-independent. **A.** SUDHL-4 cells were treated with BLyS-gel at 500 pM for 24, 48, or 72 hrs prior to collection of whole cell lysates for western blot analysis. Blots were probed using antibodies specific for caspase-9 (full-length 47 kDa; cleaved 37 kDa) and PARP (full-length 116 kDa; cleaved 89 kDa). Actin was probed as a loading control. **B.** Cells were treated with BLyS-gel at 500 pM for 48 hrs prior to analysis of caspase-3/7 activity. **C.** Cells were treated with BLyS-gel at 500 pM in the absence or presence of the general caspase inhibitor z-VAD-FMK at 10 µM. Cell viability was analyzed following 72 hrs of treatment. **D.** SUDHL-4 cells were treated with the TRAIL-R1 agonistic antibody mapatumumab at 10 µg/ml, z-VAD-FMK at 10 µM, or the combination. Cell viability was analyzed following 24 hrs of treatment. NT, no treatment; CI, caspase inhibitor.

To determine whether BLyS-gel induced protein synthesis inhibition leads to caspase-dependent apoptosis, cells were treated with BLyS-gel and caspase activation was analyzed. BLyS-gel treatment of SUDHL-4 cells induced cleavage of caspase-9 and PARP, although only a small amount of each were cleaved following 72 hrs of treatment (Fig. 4A). BLyS-gel treatment also induced moderate activation of caspase-3 & -7 (Fig. 4B). Next, cells were treated with BLyS-gel in the absence or presence of the general caspase inhibitor z-VAD-FMK. In all five cell lines tested, z-VAD-FMK failed to block the cytotoxic effects of BLyS-gel (Fig. 4C). As a control, death receptor TRAIL-R1 mediated apoptotic cell death was completely inhibited by z-VAD-FMK in SUDHL-4 cells (Fig. 4D). These results suggest BLyS-gel treatment induces moderate caspase activation, which is not required for cell death.

To further analyze the mechanism of cell death, BLyS-gel treated cells were analyzed for exposure of phosphatidylserine using annexin V (AxV). Externalization of phosphatidylserine is one of the earliest events in the apoptotic process, preceding the loss of membrane integrity. Thus, AxV and the cell impermeable dye propidium iodide (PI) are commonly used to distinguish between apoptotic (AxV^+^/PI^−^) and necrotic (AxV^+^/PI^+^) cell death. Rec-1 cells treated with BLyS-gel displayed an apoptotic phenotype with more AxV^+^/PI^−^ cells at the early time points (Fig. S2A). In contrast, SUDHL-4 cells displayed a necrotic phenotype with more AxV^+^/PI^+^ cells at all time points (Fig. S2A). A diphtheria toxin-GM-CSF fusion toxin was recently shown to induce caspase-independent “necroptosis” in target cells, which was blocked using the necroptosis inhibitor necrostatin-1 [41]. Like gelonin, diphtheria toxin kills cells by inhibiting protein synthesis. Therefore, BLyS-gel treated cells were treated with necrostatin-1 alone or in combination with z-VAD-FMK (Fig. S2B), but these conditions also failed to inhibit the cytotoxic effects BLyS-gel. Taken together, these findings suggest that BLyS-gel induces cell death by a caspase- and necroptosis-independent mechanism.

### BLyS-gel treatment activates components of the ribotoxic stress response

**Figure 5:**
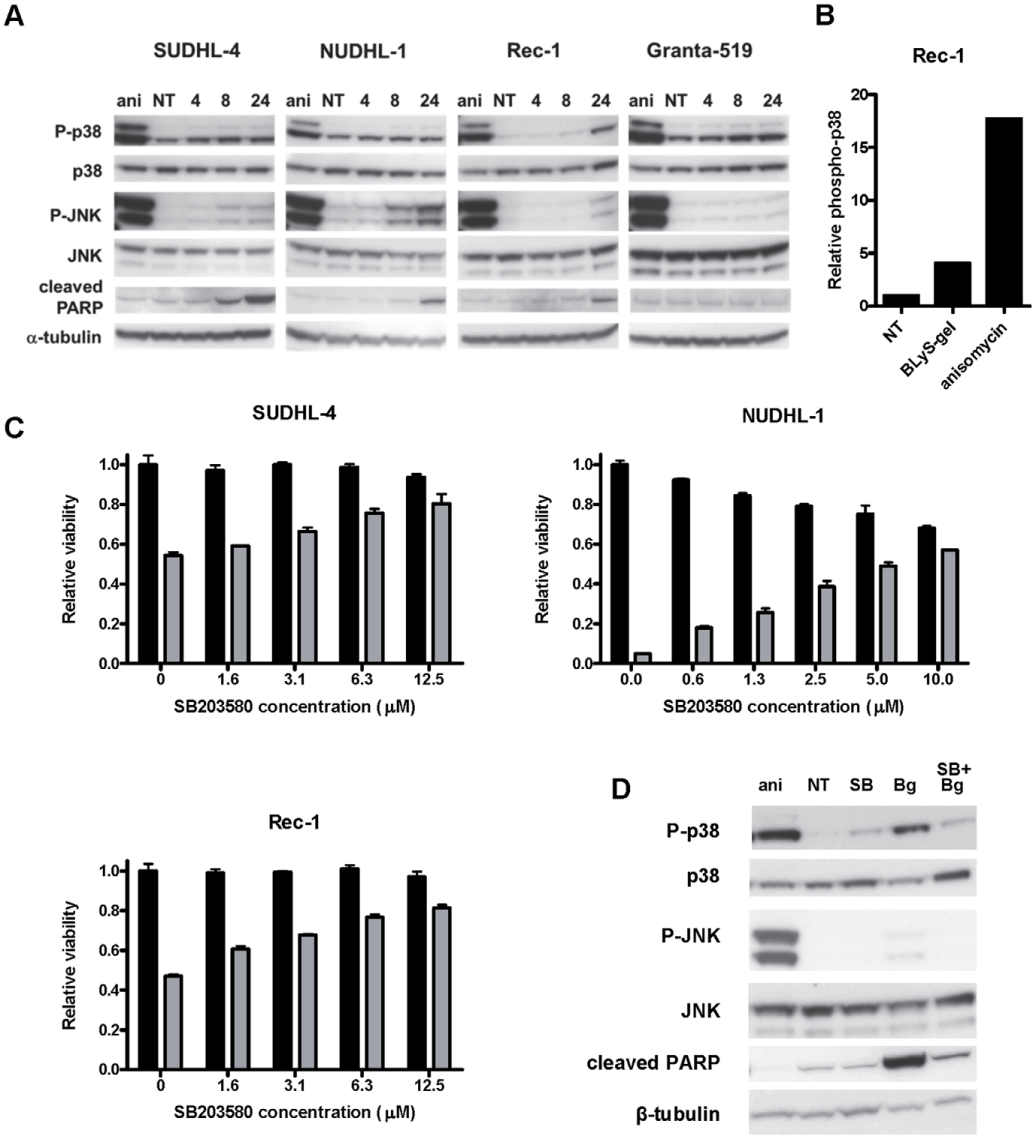
BLyS-gel treatment induces the ribotoxic-stress response. **A.** Cells were treated with BLyS-gel at 500 pM for 4, 8 or 24 hrs prior to collection of whole cell lysates for western blot analysis. Cells were also treated with anisomycin (ani) at 1 µg/ml for 1 hr as a positive control for RSR induction. Blots were probed using antibodies specific for phosphorylated and non-phosphorylated forms of p38 and JNK. Cleaved PARP was probed as a measure of cell death and α-tubulin was probed as a loading control. **B.** Cells were treated with BLyS-gel at 500 pM for 24 hrs or ani for 1 hr prior to collection of whole cell lysates. p38 phosphorylation was quantified using a quantitative ELISA assay. **C.** Cells were treated with a titer of the p38/JNK inhibitor SB203580 in the presence (gray bars) or absence (black bars) of BLyS-gel at 500 pM. Cell viability was analyzed following 72 hrs of treatment. **D.** Rec-1 cells were treated with SB203580 (SB) at 12.5 µM, BLyS-gel (Bg) at 500 pM, or the combination for 72 hrs prior to the collection of whole cell lysates for western blot analysis. Blots were probed as described in A. NT, no treatment.

Ribosome inactivating proteins and other protein synthesis inhibitors known to damage the α-sarcin/ricin loop of 28S rRNA have been shown to kill cells via induction of the “ribotoxic stress response” (RSR) [42]. This response involves activation of the p38 MAPK and JNK/SAPK signaling pathways that transmit signals required for subsequent cell death [43]. Cells treated with BLyS-gel for 4, 8, or 24 hrs were analyzed for activation of these pathways. BLyS-gel treatment induced JNK phosphorylation in BLyS-gel sensitive SUDHL-4, NUDHL-1, and Rec-1 cells, but not in the BLyS-gel insensitive Granta-519 cells (Fig. 5A). BLyS-gel treatment also induced p38 phosphorylation in the Rec-1 cells (Fig. 5A & B). The appearance of cleaved PARP corresponded with activation of JNK and/or p38 in the SUDHL-4, NUDHL-1, and Rec-1 cells, which is consistent with the low level caspase activation shown in Fig. 4A & B.

To determine whether p38 or JNK signaling was induced by binding of BLyS to BLyS receptors, Rec-1 and NUDHL-1 cells were treated with BLyS or BLyS-gel for 4, 8, and 24 hrs. BLyS-gel treatment induced p38 and JNK activation, but BLyS did not (Fig. S3), indicating p38 and JNK activation is mediated by gelonin and likely related to induction of the RSR.

### p38/JNK inhibitor reduces the cytotoxic effects of BLyS-gel

To determine whether activation of the p38 and JNK pathways contributes to BLyS-gel mediated cell death, cells were incubated with BLyS-gel in the absence or presence of the p38/JNK inhibitor SB203580. Although originally thought to be a specific inhibitor of p38 [44], SB203580 was later shown to inhibit JNK activity as well [45], [46], [47]. In all three cell lines tested, SB203580 reduced the cytotoxic effects of BLyS-gel in a dose-dependent manner (Fig. 5C). In Rec-1 cells, the effects of SB203580 on viability corresponded with a decrease in BLyS-gel-induced activation of p38 and JNK (Fig. 5D). Taken together, these data suggest that activation of p38 and JNK is at least partially responsible for mediating the cytotoxic effects of BLyS-gel.

### BLyS-gel prolongs survival of mice in xenograft models of BCP-ALL, DLBCL, and MCL

The therapeutic potential of BLyS-gel was examined using xenograft models of BCP-ALL, DLBCL, and MCL in immunodeficient mice. The Nalm-6 model of BCP-ALL is well characterized [48], [49], [50]. When injected i.v. into SCID mice, Nalm-6 cells disseminate and grow predominantly in the bone marrow, including the lower spine. This causes paralysis of the hind limbs requiring animals to be euthanized 35-45 days following challenge [51]. The Rec-1 model of MCL and NUDHL-1 model of DLBCL have not been reported in the literature previously. Intravenous injection of Rec-1 cells into SCID mice generates disseminated disease originating in the spleen and bone marrow and progressing to the lung, liver, ovary, pancreas, brain, and peripheral blood (unpublished observations). Mice ultimately become moribund, requiring sacrifice 45–55 days following injection of Rec-1 cells. Intravenous injection of NUDHL-1 cells into NSG mice generates disseminated disease that manifests in a variety of organs. Solid masses appear in lymph node regions, but the specific nodes involved differ between mice (unpublished observations). Malignant cells are also apparent in the spleen, liver, pancreas, brain, lung, ovaries, and peripheral blood at the time of sacrifice. Mice ultimately require euthanasia 50–70 days following injection of NUDHL-1 cells.

**Figure 6:**
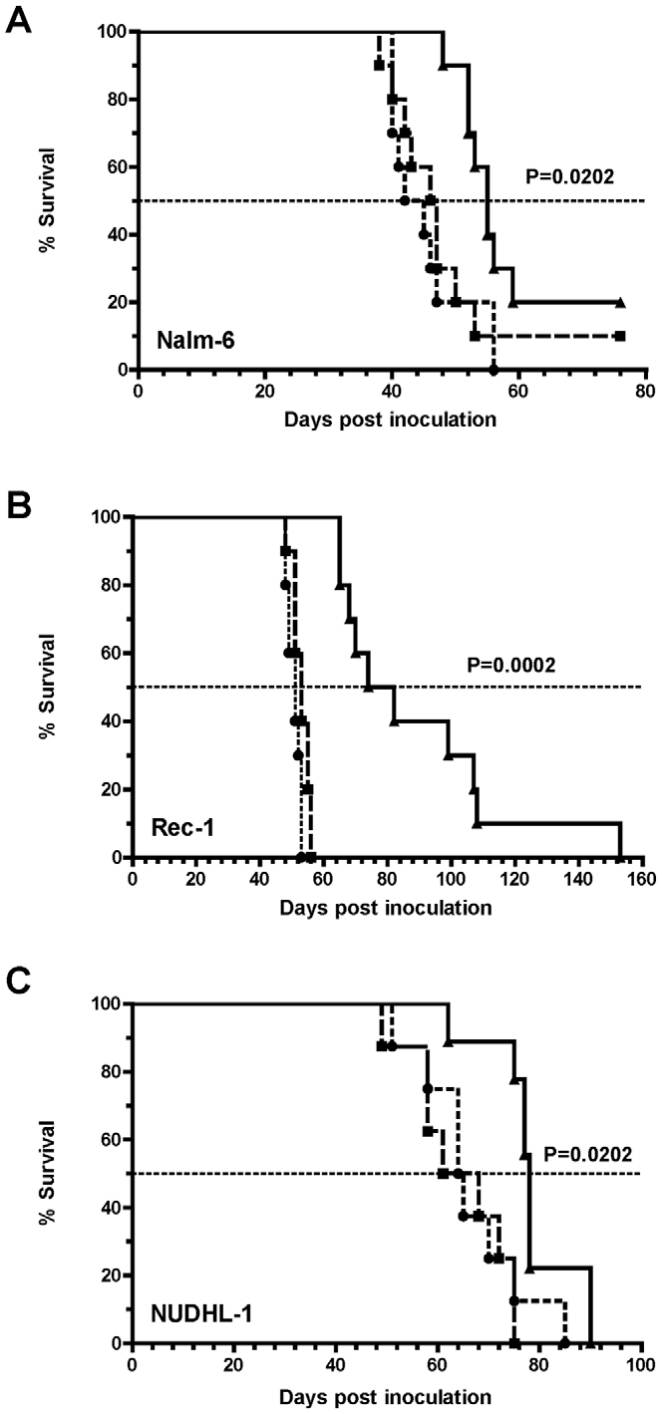
BLyS-gel prolongs survival of mice in xenograft models of BCP-ALL, MCL, and DLBCL. Nalm-6 BCP-ALL, Rec-1 MCL, or NUDHL-1 DLBCL cells (1×10^6^) were injected into the tail veins of immunodeficient mice on day 0. Mice were divided into three groups (n = 10) for i.v. treatment with free gelonin (▪), BLyS-gel (▴), or an equivalent volume of vehicle (•). All mice were injected with the murine BLyS-specific antibody 10F4 (5 mg/kg) to deplete circulating mBLyS on day 1, and treatments began on day 2. Additional 10F4 was given prior to each week of treatment. **A.** Mice injected with Nalm-6 cells were treated (2 mg/kg) 3 times per week for 2 weeks. **B.** Mice injected with Rec-1 cells were treated (2 mg/kg) 3 times per week for 2 weeks. **C.** Mice injected with NUDHL-1 cells were treated (1 mg/kg) twice per week for 4 weeks. P values refer to results of the Logrank test.

To determine whether BLyS-gel could prolong the survival of SCID mice injected with Nalm-6 or Rec-1 cells, mice were treated i.v. with 2 mg/kg BLyS-gel, free gelonin, or vehicle. Additionally, 24 hrs prior to administration of the treatments, all mice were injected with the murine BLyS-specific antibody 10F4 to deplete murine BLyS (mBLyS) from the circulation and reduce possible competition with BLyS-gel. 10F4 treatment reduced mBLyS levels in the serum at least 10-fold for up to five days (Table S2). Mice injected with Nalm-6 cells and treated with BLyS-gel for 5 consecutive days survived significantly longer (P = 0.0393) than mice receiving control treatments (Fig. S4A), although the difference in median survival between the BLyS-gel group and the control groups was modest (49 days vs. 37.5 or 42 days). In addition, BLyS-gel treated mice lost body weight, requiring one mouse to be euthanized at the end of the treatment cycle (Fig. S4B). However, the remaining mice recovered body weight quickly following the cessation of treatment. In an effort to reduce the observed BLyS-gel toxicity, the experiment was repeated using a less frequent dosing schedule of 3 times per week for 2 weeks. BLyS-gel treated mice again survived significantly longer (P = 0.0202) than controls (Fig. 6A), but without the associated body weight loss seen using the more frequent dosing schedule (Fig. S5A). Next, mice injected with Rec-1 cells were treated with BLyS-gel 3 times per week for 2 weeks. Under these conditions, BLyS-gel treated mice did not lose body weight (Fig. S5B) and survived significantly longer (P = 0.0002) than mice receiving control treatments (Fig. 6B). The difference in median survival between the BLyS-gel group and the control groups was about 25 days (77.5 days vs 51 or 53 days). NSG mice injected with NUDHL-1 cells and treated with BLyS-gel also survived significant longer (P = 0.0202) than controls (Fig. 6C). Taken together, these findings indicate BLyS-gel treatment can significantly prolong survival in multiple models of disseminated B-NHL.

### Extending the BLyS-gel treatment schedule prolongs overall survival in the Rec-1 model

**Figure 7:**
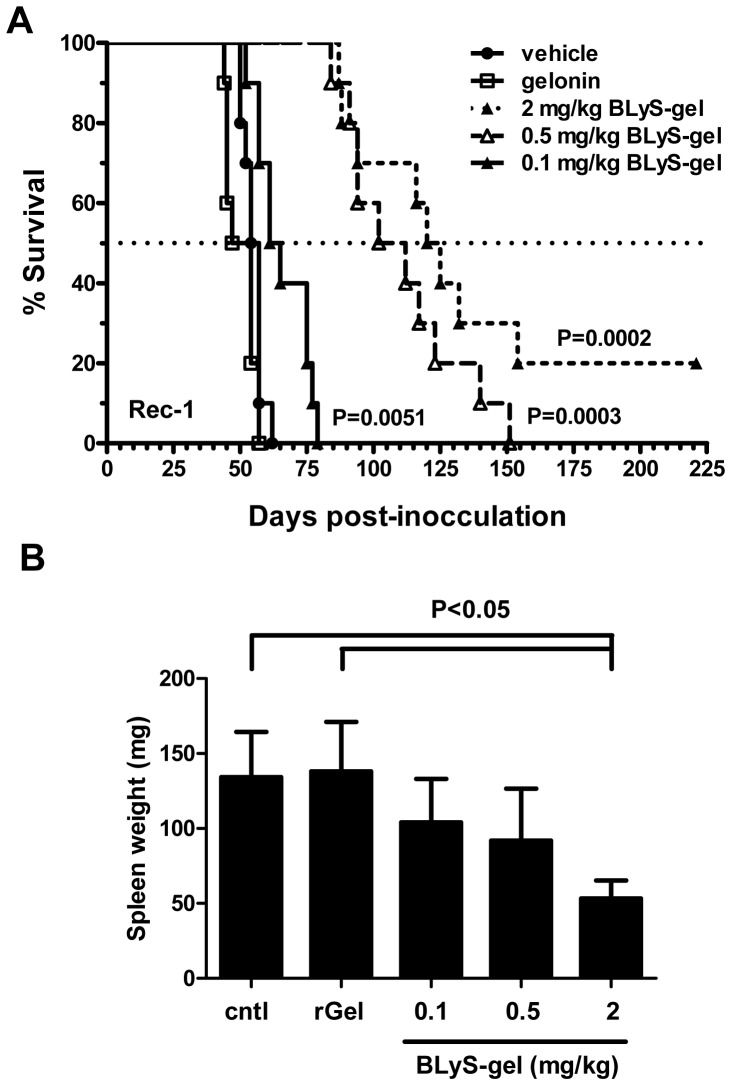
BLyS-gel inhibits MCL growth *in vivo* in a dose-dependent manner. Rec-1 cells (1×10^6^) were injected into the tail veins of SCID mice on day 0. **A.** Mice were divided into five groups (n = 10) for i.v. treatment with BLyS-gel at 2, 0.5, or 0.1 mg/kg; free gelonin at 2 mg/kg; or an equivalent volume of vehicle. All groups were injected with 10F4 at 5 mg/kg to deplete circulating mBLyS on day 1, and treatments began on day 2. Treatments were given twice per week for six weeks. Additional 10F4 was given prior to each week of treatment. P values refer to results of the Logrank test. **B.** Spleens weights at time of sacrifice. P value refers to results of the student's t test.

The Rec-1 survival experiment above was repeated to determine the effect of extending the treatment schedule from 2 weeks to 6 weeks on overall survival. BLyS-gel doses of 0.5 and 0.1 mg/kg were also tested to determine whether lower doses of BLyS-gel would be efficacious. As expected, BLyS-gel treatments prolonged survival in a dose-dependent manner (Fig. 7A). Furthermore, at the 2 mg/kg dose, median survival increased from approximately 25 days on the 2 week dosing schedule (Fig. 6B) to approximately 70 days on the 6 week dosing schedule (Fig. 7A, 123 days for BLyS-gel vs 51 or 56 days for controls). Importantly, two mice in the 2 mg/kg group were likely cured since hCD19^+^ cells were not detectable in bone marrow aspirates at the time of sacrifice on day 225 (data not shown). The 0.5 and 0.1 mg/kg doses also significantly prolonged survival (107 and 63 days; respectively), demonstrating that BLyS-gel has activity *in vivo* even at 1/20^th^ of the 2 mg/kg dose.

### BLyS-gel localizes to Rec-1 cells growing *in vivo* and dramatically reduces tumor burden in the spleen

As mentioned above, mice injected with Rec-1 cells develop a large disease burden within the spleen and bone marrow. To determine whether BLyS-gel localizes to Rec-1 cells growing these tissues, mice with terminal stage disease (5–10% body weight loss) were injected i.v. with 2 mg/kg BLyS-gel or gelonin and sacrificed four or 24 hours later. Flow cytometry was performed on homogenized spleens or bone marrow aspirates to identify the hCD19^+^ Rec-1 cells. Importantly, only hCD19^+^ cells from mice injected with BLyS-gel were also stained by an anti-gelonin antibody (Table 3), demonstrating that BLyS specifically delivers gelonin to malignant B cells *in vivo*.

**Table 3:** BLyS-gel localizes to Rec-1 cells *in vivo*.

	MFI α-gelonin/hCD19^+^ *	
Treatment	Bone Marrow	Spleen
gelonin	14.27	3.90
gelonin	13.65	-
BLyS-gel	76.86	33.89
BLyS-gel	78.04	35.46

* human CD19^+^ Rec-1 cells in bone marrow aspirates or homogenized spleens were analyzed for binding of an anti-gelonin pAb by flow cytometry as described in Material and Methods.

MFI; mean fluorescent intensity of hCD19^+^ cells stained with α-gelonin.

Given the ability of BLyS-gel to target malignant Rec-1 cells in the spleen, necropsies were performed on mice from the experiment shown in Fig. 7A to assess the effects of treatment on the spleens from these animals. Control treated mice had grossly enlarged spleens, which were completely filled with hCD20^+^ Rec-1 cells (data not shown). In contrast, the spleens of mice treated with 2 mg/kg BLyS-gel were significantly smaller (Fig. 7B) and nearly devoid of hCD20^+^ cells.

**Figure 8:**
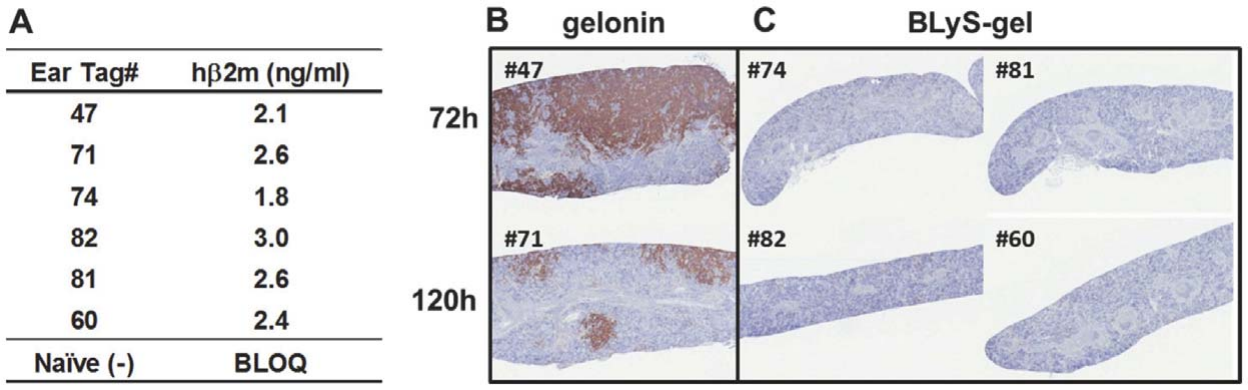
BLyS-gel eliminates MCL cells from the spleens of diseased mice. **A.** Serum samples were collected from mice 4 weeks following i.v. injection of 1×10^6^ Rec-1 cells. The concentration of human β2-microglobulin (hβ2M) was quantified by ELISA and used as a marker of established disease. Serum from a naïve mouse was used as a negative control. BLOQ; below limit of quantitation. **B & C.** Mice with established disease were treated i.v. with 2 mg/kg gelonin or BLyS-gel. Spleens were collected 72 or 120 hrs following treatment and FFPE sections were stained for human CD20^+^ Rec-1 cells (reddish brown).

To determine whether BLyS-gel could reduce disease burden in the spleens of mice with “established” disease, mice were injected i.v. with Rec-1 cells. Human β2-microglobulin (hβ2M) has been used to monitor progression of disseminated disease in xenograft models [33], and preliminary studies indicated that hβ2M was detectable in serum four weeks after of injection of Rec-1 cells (Table S3). Detection of hβ2M in the serum of mice 25 days after cell injection confirmed the presence of established disease in six mice (Fig. 8A). These mice were then treated with gelonin or BLyS-gel at 2 mg/kg and spleens were collected 72 or 120 hrs later for analysis of disease burden by immunohistochemistry. At both time points, hCD20^+^ cells were clearly visible in the spleens of gelonin treated mice (Fig. 8B). In contrast, hCD20^+^ cells were completely eradicated from the spleens of BLyS-gel treated mice (Fig. 8C). These results indicate that established disease within the spleen is effectively cleared 72 hrs following a single dose of BLyS-gel in a novel and aggressive model of MCL.

## Discussion

The objective of the current study was to determine the efficacy of using BLyS as a targeting agent for the delivery of a cytotoxic “payload,” such as gelonin, to malignant B cells. A panel of over 40 B cell NHL cell lines of various subtypes was screened for BLyS receptor expression and sensitivity to BLyS-gel mediated cytotoxicity. At least one of the three BLyS receptors was detected on nearly every malignant B cell line tested and BLyS-gel treatment reduced the viability of a number of these cell lines. Interestingly, sensitivity to BLyS-gel treatment was generally restricted to the MCL, DLBCL, and BCP-ALL subtypes, while the B-CLL, BL and MM subtypes were insensitive. The preferential sensitivity of MCL, DLBCL, and BCP-ALL cell lines to a similar BLyS-gelonin fusion toxin (rGel/BLyS) was reported previously [27], [52]. The insensitivity of B-CLL cells lines to BLyS-gel treatment seems to conflict with an earlier report demonstrating that rGel/BLyS is cytotoxic to primary B-CLL lymphocytes freshly isolated from patient blood [29]. Therefore, CD19^+^ cells isolated from the blood of three B-CLL patients were thawed and tested for sensitivity to BLyS-gel. BLyS-gel treatment had no cytotoxic effects on these cells (Fig. S6A), despite cell surface expression of BR3 (Fig. S6B). Importantly, B-CLL cells from these patients were capable of binding BLyS and internalizing BLyS-gel (Fig. S6C). The difference in B-CLL cell sensitivity to BLyS-gel may be due to i) the use of annexin V to measure cytotoxicity in the prior study, or ii) increased sensitivity of fresh primary B-CLL cells relative to the frozen B-CLL cell lines or primary B-CLL cells used here. Additional studies on primary malignant B cells may help to resolve this issue.

For gelonin to induce cell death, it must be internalized by target cells and released into the cytoplasm [17]. Internalization studies indicate that BLyS-gel is internalized by all cells expressing BLyS receptors, yet some cell lines remain resistant to the cytotoxic effects of BLyS-gel. These findings suggest that a failure of BLyS-gel to enter the cytoplasm following internalization is a likely cause of resistance to BLyS-gel mediated cytotoxicity. This is a common problem for fusion toxins, where endo-lysosomal sequestration and degradation is often a major obstacle for successful drug delivery [53]. Importantly, use of the endosomotropic drug chloroquine enhanced the cytotoxic effects of BLyS-gel on several resistant cells lines, supporting the notion that endosomal entrapment and/or rapid degradation in the lysosomes is a likely mechanism of BLyS-gel resistance.

To determine whether the cytotoxic effects of rGel/BLyS were mediated by BLyS receptors, Lyu et. al. used soluble BLyS receptor-Fc fusion constructs [27], [28]. As expected, these constructs inhibited the cytotoxicity of rGel/BLyS. This approach demonstrated that soluble BLyS receptors can bind rGel/BLyS and compete for binding to cell surface BLyS receptors. However, this method did not identify which BLyS receptors were responsible for mediating rGel/BLyS cytotoxicity in cells. To address this issue, BLyS receptor blocking antibodies were used here to demonstrate that no single BLyS receptor is responsible for mediating the cytotoxic effects of BLyS-gel. In the four cell lines examined, BR3 and TACI combined to mediate most or all of the BLyS-gel cytotoxicity, while the contribution of BCMA was minimal. Moreover, the affinity of BLyS for BCMA is lower than for BR3 and TACI [6], and no cell lines expressing BCMA alone were sensitive to BLyS-gel. Thus, BR3 and TACI are the likely mediators of BLyS-gel cytotoxicity, although a role for BCMA cannot be completely excluded based on the small number of cell lines examined.

BLyS-gel treatment inhibited protein synthesis in BLyS-gel sensitive cell lines, which is consistent with the passive mechanism of cell death generally considered the primary means of RIP-mediated cell killing [54]. More recent findings suggest that RIPs may also actively induce programmed cell death through multiple mechanisms [43], [55]. In this regard, BLyS-gel treatment was shown to induce moderate caspase activation and PARP cleavage, which are hallmarks of the apoptotic pathway. However, treatment with z-VAD-FMK did not inhibit BLyS-gel mediated cytotoxicity in any of the cell lines tested, suggesting the mechanism of action is caspase-independent. This contrasts with results reported by Lyu et al, which showed that the effects of rGel/BLyS were inhibited by z-VAD-FMK, though in those studies z-VAD-FMK was used at significantly higher concentrations than used here [27]. A number of caspase-independent cell death mechanisms are known, some of which involve the p38 MAPK and JNK/SAPK signaling pathways [56], [57]. More specifically, RIPs have been shown to kill cells via induction of the “ribotoxic stress response” (RSR) [42]. This response involves activation of the p38 MAPK and JNK/SAPK signaling pathways that transmit signals required for subsequent cell death [43]. Importantly, p38 and/or JNK signaling pathways were activated in BLyS-gel sensitive cell lines, and were inhibited by the p38/JNK inhibitor SB203580. Treatment with SB203580 also reduced BLyS-gel induced cytotoxicity suggesting that activation of the RSR has a major role mediating the cytotoxic effects of BLyS-gel. Other studies found that rGel/BLyS induced cell death of the activated B cell (ABC) subtype of DLBCL was dependent upon disruption of other signaling pathways, such as NF-κB, Stat3 and IL-6R [27], [28], [30]. Whether activation of the RSR affects these pathways in ABC-DLBCL cells is unknown.

BLyS-gel treatment prolonged the survival of mice in three xenograft models of disseminated B NHL disease. BCP-ALL develops by transformation of normal B cell progenitors in the bone marrow, which do not express BLyS receptors [8]; therefore, the recent discovery of BR3 on BCP-ALLs was somewhat unexpected [12], [13]. The cell surface expression of BR3 by BCP-ALL cell lines was confirmed here and BLyS-gel treatment significantly prolonged the survival of mice injected with Nalm-6 BCP-ALL cells. Importantly, these findings are consistent with a recent report demonstrating the therapeutic effects of rGel/BLyS treatment using disseminated xenograft models established with patient derived BCP-ALL cells [52].

To the authors' knowledge, this is the first report to describe the use of NUDHL-1 DLBCL and Rec-1 MCL cell lines to establish disseminated models of disease in immunodeficient mice. This is also the first report to demonstrate that BLyS-gel treatment prolongs the survival of mice with disseminated DLBCL and MCL disease. BLyS-gel treatment extended survival in the Rec-1 MCL model in a dose-dependent manner, with a median survival increase of over 70 days relative to controls at the highest dose. BLyS-gel was also shown bind to Rec-1 MCL cells growing in the bone marrow and spleen, and mice treated with BLyS-gel had smaller spleens due to a reduction in tumor burden within this organ. Furthermore, BLyS-gel was shown to eradicate established disease within the spleen only 72 hrs following a single injection. However, treatment of mice with established Rec-1 disease (verified by serum hβ2M) did not significantly prolong survival (data not shown). Thus, although BLyS-gel treatment efficiently eliminates disease within the spleen, established disease within other organs remained refractory to BLyS-gel in this model. It is not uncommon for organ specific niches to protect cancer cells from the cytotoxic effects of targeted therapy. In this regard, rGel/BLyS was recently shown to eliminate circulating cancer cells in a mouse model of disseminated BCP-ALL, but had little effect on cancer cells in the bone marrow unless these cells were mobilized using a CXCR4 antagonist [52]. It is possible that similar rational combination strategies could enhance the effects of BLyS-gel treatment in MCL models of established disease.

Given the ability of clq to enhance the *in vitro* cytotoxicity of BLyS-gel, one could consider using clq to enhance the *in vivo* efficacy of BLyS-gel as well. However, previous attempts to use clq to enhance the efficacy of immunotoxins *in vivo* have failed, likely because the concentrations required for the endo-lysosomotropic effects of clq are too high for *in vivo* studies. Roth et al have reported that co-administration of clq with an immunotoxin failed to enhance the activity *in vivo* [58]. In a more rigorous evaluation of a CD22 directed immunotoxin, Van Horssen et al came to a similar conclusion [59]. These authors generated a sustained concentration of clq in mice by implanting a mini-pump, but found the maximally tolerated serum concentration (3.9 µM) was too low to be effective. In this regard, 100 µM clq is required to enhance the cytotoxic effects of BLyS-gel in the *in vitro* studies presented here.

In summary, these studies demonstrate that the BLyS-gel fusion toxin is highly cytotoxic to B cell NHLs expressing BLyS receptors, especially the MCL, DLBCL, and BCP-ALL subtypes. BLyS-gel treatment inhibits protein synthesis in target cells and induces caspase-independent cell death that is largely mediated by activation of the RSR. BLyS-gel also significantly prolongs the survival of mice in xenograft models of BCP-ALL, DLBCL, and MCL. Together, these findings suggest BLyS has significant potential as a targeting ligand for the delivery of cytotoxic “payloads” to malignant B cells.

## Supporting Information

Materials and Methods S1 Descriptions for procedures that were used to collect data displayed in supporting information.
(DOC)

Figure S1 **Characterization of BLyS-gel. A.** Purified BLyS-gel was analyzed by SDS-PAGE under reducing (R) and non-reducing (NR) conditions. Gelcode Blue staining revealed a single band of approximately 45 kD (left panel), consistent with the expected size of BLyS-gel. Purified BLyS-gel or recombinant human BLyS were analyzed by western blot with anti-gelonin or anti-BLyS antibodies (right panels). Molecular weights (kD) are indicated to the left of each panel. **B.** Flow cytometric analysis of BLyS-gel binding to B & T cell lines. B cell lines SUDHL-4, IM-9, Jeko-1 and Mino all express at least one BLyS receptor (Fig. 1A and Table 1). The Jurkat T cell line, which does not express BLyS receptors, was used a negative control. Gray-shaded peak, gelonin detection reagents only; dashed line, gelonin+detection reagents; solid line, BLyS-gel+detection reagents. **C.** BLyS competes for binding of BLyS-gel to B cells. SUDHL-4 cells were incubated with 20 µg/ml BLyS-gel along with a titer of recombinant human BLyS from 80 ng/ml to 20 µg/ml. BLyS-gel binding was then analyzed by flow cytometry using an anti-gelonin antibody. Data are presented as the mean fluorescence intensity (MFI) of anti-gelonin stained cells.
(PDF)

Figure S2 **BLyS-gel treatment induces markers of apoptotic and necrotic cell death, but cytotoxicity is not blocked by caspase or necroptosis inhibitors.** **A.** Rec-1 or SUDHL-4 cells were treated with BLyS-gel at 500 pM 0, 24, 48, or 72 hrs. Cells were then stained for phosphatidylserine exposure using annexin V and for membrane integrity using propidium iodide and analyzed by flow cytometry. The percentage of cells AxV^−^/PI^−^ (viable), AxV^+^/PI^+^ (necrotic/dead) and AxV^+^/PI^−^ (apoptotic) is shown in each quadrant. **B.** Rec-1 or Nalm-6 cells were treated with the indicated combinations BLyS-gel (Bg) at 500 pM, the general caspase inhibitor z-VAD-FMK (CI) at 10 µM, or the necroptosis inhibitor necrostatin-1 (N) at 10 µM. Cell viability was analyzed following 72 hrs of treatment. Data are presented as viability relative to untreated cells. NT, no treatment.
(PDF)

Figure S3 **BLyS-gel, but not BLyS, induces p38 and JNK phosphorylation.** Rec-1 or NUDHL-1 cells were treated with BLyS-gel or BLyS at 500 pM for 4, 8 or 24 hrs prior to collection of whole cell lysates for western blot analysis. Cells were also treated with anisomycin (ani) as a positive control for induction of p38 and JNK phosphorylation. Blots were probed using antibodies specific for phosphorylated and non-phosphorylated forms of p38 or JNK. α-tubulin was probed as a loading control.
(PDF)

Figure S4 **BLyS-gel treatment effects on survival and body weight in the Nalm-6 model of BCP-ALL.** Nalm-6 BCP-ALL cells (1×10^6^) were injected into the tail veins of SCID mice on day 0. Mice were divided into three groups (n = 10) for i.v. treatment with free gelonin, BLyS-gel, or an equivalent volume of vehicle. All mice were injected with the murine BLyS-specific antibody 10F4 (5 mg/kg) to deplete circulating mBLyS on day 1, and treatments began on day 2. Mice were treated (2 mg/kg) on days 2–6. **A.** Kaplan-Meyer survival curve. P value refers to results of the Logrank test. **B.** Percent body weight change. On day 6, one of the BLyS-gel treated animals died as result of apparent treatment related toxicity, reducing the number of mice in this group to 9. Arrow indicates the last day of treatment.
(PDF)

Figure S5 **BLyS-gel treatment effects on body weight in various models.** Nalm-6 BCP-ALL, Rec-1 MCL, or NUDHL-1 DLBCL cells (1×10^6^) were injected into the tail veins of immunodeficient mice on day 0. Mice were divided into three groups (n = 10) for i.v. treatment with free gelonin, BLyS-gel, or an equivalent volume of vehicle. All mice were injected with the murine BLyS-specific antibody 10F4 (5 mg/kg) to deplete circulating mBLyS on day 1, and treatments began on day 2 in all studies. Arrows indicate the last day of treatment. **A.** Mice were treated (2 mg/kg) 3 times per week for 2 weeks. **B.** Mice were treated (2 mg/kg) 3 times per week for 2 weeks. **C.** Mice were treated (1 mg/kg) twice per week for 4 weeks. **D.** Mice were treated (2 mg/kg) twice per week for 6 weeks.
(PDF)

Figure S6 **BLyS-gel effects on primary B-CLL cells. A.** CD19^+^ cells isolated from the blood of three B-CLL patients were thawed and treated with a titer of BLyS-gel, gelonin, or bortezomib. Cell viability was analyzed following 72 hrs of treatment. Data are presented as viability relative to untreated cells. NT, no treatment. **B.** Primary B-CLL cells were analyzed for BLyS receptor expression by flow cytometry. Samples were stained with antibodies specific for BCMA, TACI or BR3. Alternatively, the ability of cells to bind BLyS was determined by incubation with biotinylated BLyS. Values shown represent the mean fluorescence intensity (MFI). **C.** BLyS-gel internalization into primary B-CLL cells was analyzed by flow cytometry. Samples were incubated with BLyS-gel for 30 min at 4°C, then transferred to 37°C for 30 to 120 min to allow BLyS-gel to internalize. The remaining surface bound BLyS-gel was then detected using a gelonin-specific antibody. The decrease in MFI over time at 37°C indicates internalization has occurred.
(PDF)

Table S1 **BLyS-gel binds BLyS-receptors.** Affinity data for BLyS-gel binding to BLyS-receptors determined by surface plasmon resonance.
(PDF)

Table S2 **mBLyS concentration in blood of SCID mice.** Quantification of murine BLyS levels before and after injection of the mBLyS-specific antibody 10F4.
(PDF)

Table S3 **hβ2M concentration in mouse serum.** Quantification of human β2M levels in serum of mice inoculated with Rec-1 cells.
(PDF)

## References

[1] Nardelli B, Belvedere O, Roschke V, Moore PA, Olsen HS, et al (2001) Synthesis and release of B-lymphocyte stimulator from myeloid cells. Blood 97: 198–204. 11133761 10.1182/blood.v97.1.198

[2] Scapini P, Nardelli B, Nadali G, Calzetti F, Pizzolo G, et al (2003) G-CSF-stimulated neutrophils are a prominent source of functional BLyS. J Exp Med 197: 297–302. 12566413 10.1084/jem.20021343 PMC2193843

[3] Tai YT, Li XF, Breitkreutz I, Song W, Neri P, et al (2006) Role of B-cell-activating factor in adhesion and growth of human multiple myeloma cells in the bone marrow microenvironment. Cancer Res 66: 6675–6682. 16818641 10.1158/0008-5472.CAN-06-0190

[4] Mackay F, Schneider P (2009) Cracking the BAFF code. Nat Rev Immunol 9: 491–502. 19521398 10.1038/nri2572

[5] Day ES, Cachero TG, Qian F, Sun Y, Wen D, et al (2005) Selectivity of BAFF/BLyS and APRIL for binding to the TNF family receptors BAFFR/BR3 and BCMA. Biochemistry 44: 1919–1931. 15697217 10.1021/bi048227k

[6] Bossen C, Schneider P (2006) BAFF, APRIL and their receptors: structure, function and signaling. Semin Immunol 18: 263–275. 16914324 10.1016/j.smim.2006.04.006

[7] Dillon SR, Gross JA, Ansell SM, Novak AJ (2006) An APRIL to remember: novel TNF ligands as therapeutic targets. Nat Rev Drug Discov 5: 235–246. 16474316 10.1038/nrd1982

[8] Treml JF, Hao Y, Stadanlick JE, Cancro MP (2009) The BLyS family: toward a molecular understanding of B cell homeostasis. Cell Biochem Biophys 53: 1–16. 19034695 10.1007/s12013-008-9036-1 PMC2654184

[9] Novak AJ, Grote DM, Stenson M, Ziesmer SC, Witzig TE, et al (2004) Expression of BLyS and its receptors in B-cell non-Hodgkin lymphoma: correlation with disease activity and patient outcome. Blood 104: 2247–2253. 15251985 10.1182/blood-2004-02-0762

[10] Novak AJ, Darce JR, Arendt BK, Harder B, Henderson K, et al (2004) Expression of BCMA, TACI, and BAFF-R in multiple myeloma: a mechanism for growth and survival. Blood 103: 689–694. 14512299 10.1182/blood-2003-06-2043

[11] Nakamura N, Hase H, Sakurai D, Yoshida S, Abe M, et al (2005) Expression of BAFF-R (BR 3) in normal and neoplastic lymphoid tissues characterized with a newly developed monoclonal antibody. Virchows Arch 447: 53–60. 16025281 10.1007/s00428-005-1275-6

[12] Onda K, Iijima K, Katagiri YU, Okita H, Saito M, et al (2010) Differential effects of BAFF on B cell precursor acute lymphoblastic leukemia and Burkitt lymphoma. Int J Hematol 91: 808–819. 20428981 10.1007/s12185-010-0567-z

[13] Parameswaran R, Muschen M, Kim YM, Groffen J, Heisterkamp N (2010) A functional receptor for B-cell-activating factor is expressed on human acute lymphoblastic leukemias. Cancer Res 70: 4346–4356. 20460528 10.1158/0008-5472.CAN-10-0300 PMC2880197

[14] Haiat S, Billard C, Quiney C, Ajchenbaum-Cymbalista F, Kolb JP (2006) Role of BAFF and APRIL in human B-cell chronic lymphocytic leukaemia. Immunology 118: 281–292. 16827889 10.1111/j.1365-2567.2006.02377.x PMC1782305

[15] Briones J, Timmerman JM, Hilbert DM, Levy R (2002) BLyS and BLyS receptor expression in non-Hodgkin's lymphoma. Exp Hematol 30: 135–141. 11823048 10.1016/s0301-472x(01)00774-3

[16] Ansell SM, Armitage J (2005) Non-Hodgkin lymphoma: diagnosis and treatment. Mayo Clin Proc 80: 1087–1097. 16092591 10.4065/80.8.1087

[17] Kreitman RJ (2009) Recombinant immunotoxins containing truncated bacterial toxins for the treatment of hematologic malignancies. BioDrugs 23: 1–13. 19344187 10.2165/00063030-200923010-00001 PMC2671643

[18] Kreitman RJ, Squires DR, Stetler-Stevenson M, Noel P, FitzGerald DJ, et al (2005) Phase I trial of recombinant immunotoxin RFB4(dsFv)-PE38 (BL22) in patients with B-cell malignancies. J Clin Oncol 23: 6719–6729. 16061911 10.1200/JCO.2005.11.437

[19] Kreitman RJ, Stetler-Stevenson M, Margulies I, Noel P, Fitzgerald DJ, et al (2009) Phase II trial of recombinant immunotoxin RFB4(dsFv)-PE38 (BL22) in patients with hairy cell leukemia. J Clin Oncol 27: 2983–2990. 19414673 10.1200/JCO.2008.20.2630 PMC2702232

[20] Kreitman RJ, Wilson WH, Bergeron K, Raggio M, Stetler-Stevenson M, et al (2001) Efficacy of the anti-CD22 recombinant immunotoxin BL22 in chemotherapy-resistant hairy-cell leukemia. N Engl J Med 345: 241–247. 11474661 10.1056/NEJM200107263450402

[21] Veenendaal LM, Jin H, Ran S, Cheung L, Navone N, et al (2002) In vitro and in vivo studies of a VEGF121/rGelonin chimeric fusion toxin targeting the neovasculature of solid tumors. Proc Natl Acad Sci U S A 99: 7866–7871. 12060733 10.1073/pnas.122157899 PMC122986

[22] Ran S, Mohamedali KA, Luster TA, Thorpe PE, Rosenblum MG (2005) The vascular-ablative agent VEGF(121)/rGel inhibits pulmonary metastases of MDA-MB-231 breast tumors. Neoplasia 7: 486–496. 15967101 10.1593/neo.04631 PMC1501168

[23] Joshi BH, Kawakami K, Leland P, Puri RK (2002) Heterogeneity in interleukin-13 receptor expression and subunit structure in squamous cell carcinoma of head and neck: differential sensitivity to chimeric fusion proteins comprised of interleukin-13 and a mutated form of Pseudomonas exotoxin. Clin Cancer Res 8: 1948–1956. 12060640

[24] Nardelli B, Moore PA, Li Y, Hilbert DM (2002) B lymphocyte stimulator (BLyS): a therapeutic trichotomy for the treatment of B lymphocyte diseases. Leuk Lymphoma 43: 1367–1373. 12389615 10.1080/10428190290033297

[25] Riccobene TA, Miceli RC, Lincoln C, Knight Y, Meadows J, et al (2003) Rapid and specific targeting of 125I-labeled B lymphocyte stimulator to lymphoid tissues and B cell tumors in mice. J Nucl Med 44: 422–433. 12621010

[26] Belch A, McEwan A, Hewitt J, Riauka T, Stabin M, et al (2004) Tumor Targeting, Dosimetry and Clinical Response Data for Lymphorad-131 (LR131; Iodine I-131 Labeled B-Lymphocyte Stimulator) in Patients with Relapsed/Refractory Non-Hodgkin's Lymphoma. ASH Annual Meeting Abstracts 104: 750.

[27] Lyu MA, Cheung LH, Hittelman WN, Marks JW, Aguiar RC, et al (2007) The rGel/BLyS fusion toxin specifically targets malignant B cells expressing the BLyS receptors BAFF-R, TACI, and BCMA. Mol Cancer Ther 6: 460–470. 17267661 10.1158/1535-7163.MCT-06-0254

[28] Lyu MA, Rai D, Ahn KS, Sung B, Cheung LH, et al (2010) The rGel/BLyS fusion toxin inhibits diffuse large B-cell lymphoma growth in vitro and in vivo. Neoplasia 12: 366–375. 20454508 10.1593/neo.91960 PMC2864474

[29] Nimmanapalli R, Lyu MA, Du M, Keating MJ, Rosenblum MG, et al (2007) The growth factor fusion construct containing B-lymphocyte stimulator (BLyS) and the toxin rGel induces apoptosis specifically in BAFF-R-positive CLL cells. Blood 109: 2557–2564. 17119117 10.1182/blood-2006-08-042424

[30] Lyu MA, Sung B, Cheung LH, Marks JW, Aggarwal BB, et al (2010) The rGel/BLyS fusion toxin inhibits STAT3 signaling via down-regulation of interleukin-6 receptor in diffuse large B-cell lymphoma. Biochem Pharmacol 80: 1335–1342. 20654581 10.1016/j.bcp.2010.07.017

[31] Stirpe F, Olsnes S, Pihl A (1980) Gelonin, a new inhibitor of protein synthesis, nontoxic to intact cells. Isolation, characterization, and preparation of cytotoxic complexes with concanavalin A. J Biol Chem 255: 6947–6953. 7391060

[32] Barbieri L, Battelli MG, Stirpe F (1993) Ribosome-inactivating proteins from plants. Biochim Biophys Acta 1154: 237–282. 8280743 10.1016/0304-4157(93)90002-6

[33] Wang M, Zhang L, Han X, Yang J, Qian J, et al (2008) A severe combined immunodeficient-hu in vivo mouse model of human primary mantle cell lymphoma. Clin Cancer Res 14: 2154–2160. 18381957 10.1158/1078-0432.CCR-07-4409

[34] Oren DA, Li Y, Volovik Y, Morris TS, Dharia C, et al (2002) Structural basis of BLyS receptor recognition. Nat Struct Biol 9: 288–292. 11862220 10.1038/nsb769

[35] Kanakaraj P, Migone TS, Nardelli B, Ullrich S, Li Y, et al (2001) BLyS binds to B cells with high affinity and induces activation of the transcription factors NF-kappaB and Elf-1. Cytokine 13: 25–31. 11145839 10.1006/cyto.2000.0793

[36] Endo T, Nishio M, Enzler T, Cottam HB, Fukuda T, et al (2007) BAFF and APRIL support chronic lymphocytic leukemia B-cell survival through activation of the canonical NF-kappaB pathway. Blood 109: 703–710. 16973958 10.1182/blood-2007-04-081786 PMC1890820

[37] Parameswaran R, Muschen M, Kim YM, Groffen J, Heisterkamp N A functional receptor for B-cell-activating factor is expressed on human acute lymphoblastic leukemias. Cancer Res 70: 4346–4356. 20460528 10.1158/0008-5472.CAN-10-0300 PMC2880197

[38] Pirie CM, Hackel BJ, Rosenblum MG, Wittrup KD (2011) Convergent potency of internalized gelonin immunotoxins across varied cell lines, antigens, and targeting moieties. J Biol Chem 286: 4165–4172. 21138845 10.1074/jbc.M110.186973 PMC3039404

[39] Varkouhi AK, Scholte M, Storm G, Haisma HJ (2011) Endosomal escape pathways for delivery of biologicals. J Control Release 151: 220–228. 21078351 10.1016/j.jconrel.2010.11.004

[40] Mahmud H, Dalken B, Wels WS (2009) Induction of programmed cell death in ErbB2/HER2-expressing cancer cells by targeted delivery of apoptosis-inducing factor. Mol Cancer Ther 8: 1526–1535. 19509241 10.1158/1535-7163.MCT-08-1149

[41] Horita H, Frankel AE, Thorburn A (2008) Acute myeloid leukemia-targeted toxin activates both apoptotic and necroptotic death mechanisms. PLoS One 3: e3909. 19079542 10.1371/journal.pone.0003909 PMC2592546

[42] Iordanov MS, Pribnow D, Magun JL, Dinh TH, Pearson JA, et al (1997) Ribotoxic stress response: activation of the stress-activated protein kinase JNK1 by inhibitors of the peptidyl transferase reaction and by sequence-specific RNA damage to the alpha-sarcin/ricin loop in the 28S rRNA. Mol Cell Biol 17: 3373–3381. 9154836 10.1128/mcb.17.6.3373 PMC232190

[43] Narayanan S, Surendranath K, Bora N, Surolia A, Karande AA (2005) Ribosome inactivating proteins and apoptosis. FEBS Lett 579: 1324–1331. 15733836 10.1016/j.febslet.2005.01.038

[44] Cuenda A, Rouse J, Doza YN, Meier R, Cohen P, et al (1995) SB 203580 is a specific inhibitor of a MAP kinase homologue which is stimulated by cellular stresses and interleukin-1. FEBS Lett 364: 229–233. 7750577 10.1016/0014-5793(95)00357-f

[45] Clerk A, Sugden PH (1998) The p38-MAPK inhibitor, SB203580, inhibits cardiac stress-activated protein kinases/c-Jun N-terminal kinases (SAPKs/JNKs). FEBS Lett 426: 93–96. 9598985 10.1016/s0014-5793(98)00324-x

[46] Whitmarsh AJ, Yang SH, Su MS, Sharrocks AD, Davis RJ (1997) Role of p38 and JNK mitogen-activated protein kinases in the activation of ternary complex factors. Mol Cell Biol 17: 2360–2371. 9111305 10.1128/mcb.17.5.2360 PMC232085

[47] Harada J, Sugimoto M (1999) An inhibitor of p38 and JNK MAP kinases prevents activation of caspase and apoptosis of cultured cerebellar granule neurons. Jpn J Pharmacol 79: 369–378. 10230866 10.1254/jjp.79.369

[48] Shah SA, Halloran PM, Ferris CA, Levine BA, Bourret LA, et al (1993) Anti-B4-blocked ricin immunotoxin shows therapeutic efficacy in four different SCID mouse tumor models. Cancer Res 53: 1360–1367. 7680284

[49] Brentjens RJ, Santos E, Nikhamin Y, Yeh R, Matsushita M, et al (2007) Genetically targeted T cells eradicate systemic acute lymphoblastic leukemia xenografts. Clin Cancer Res 13: 5426–5435. 17855649 10.1158/1078-0432.CCR-07-0674

[50] Herrera L, Yarbrough S, Ghetie V, Aquino DB, Vitetta ES (2003) Treatment of SCID/human B cell precursor ALL with anti-CD19 and anti-CD22 immunotoxins. Leukemia 17: 334–338. 12592332 10.1038/sj.leu.2402790

[51] Bendall LJ, Nilsson SK, Khan NI, James A, Bonnet C, et al (2004) Role of CD44 variant exon 6 in acute lymphoblastic leukaemia: association with altered bone marrow localisation and increased tumour burden. Leukemia 18: 1308–1311. 15152268 10.1038/sj.leu.2403393

[52] Parameswaran R, Yu M, Lyu MA, Lim M, Rosenblum MG, et al. (2012) Treatment of acute lymphoblastic leukemia with an rGel/BLyS fusion toxin. Leukemia. 10.1038/leu.2012.54 PMC3376225 22373785

[53] Weldon JE, Xiang L, Chertov O, Margulies I, Kreitman RJ, et al (2009) A protease-resistant immunotoxin against CD22 with greatly increased activity against CLL and diminished animal toxicity. Blood 113: 3792–3800. 18988862 10.1182/blood-2008-08-173195 PMC2670794

[54] Stirpe F (2004) Ribosome-inactivating proteins. Toxicon 44: 371–383. 15302521 10.1016/j.toxicon.2004.05.004

[55] Polito L, Bortolotti M, Farini V, Battelli MG, Barbieri L, et al (2009) Saporin induces multiple death pathways in lymphoma cells with different intensity and timing as compared to ricin. Int J Biochem Cell Biol 41: 1055–1061. 18935973 10.1016/j.biocel.2008.09.021

[56] Broker LE, Kruyt FA, Giaccone G (2005) Cell death independent of caspases: a review. Clin Cancer Res 11: 3155–3162. 15867207 10.1158/1078-0432.CCR-04-2223

[57] Wagner EF, Nebreda AR (2009) Signal integration by JNK and p38 MAPK pathways in cancer development. Nat Rev Cancer 9: 537–549. 19629069 10.1038/nrc2694

[58] Roth JA, Ames RS, Fry K, Lee HM, Scannon PJ (1988) Mediation of reduction of spontaneous and experimental pulmonary metastases by ricin A-chain immunotoxin 45-2D9-RTA with potentiation by systemic monensin in mice. Cancer Res 48: 3496–3501. 3259469

[59] Van Horssen PJ, Preijers FW, Van Oosterhout YV, Eling WM, De Witte T (2000) Relationship of the CD22 immunotoxin dose and the tumour establishment in a SCID mice model. Leuk Lymphoma 39: 591–599. 11342342 10.3109/10428190009113389

