# Supplementary material for: Fusion Toxin BLyS-Gelonin Inhibits Growth of Malignant Human B Cell Lines In Vitro and In Vivo
Source: PLoS One. 2012 Oct 9;7(10):e47361. doi: 10.1371/journal.pone.0047361 (PMC3467252; doi:10.1371/journal.pone.0047361)
Supplement: Materials and Methods S1 — Descriptions for procedures that were used to collect data displayed in supporting information. (DOC) [file pone.0047361.s001.doc]

**Supplementary information**

**MATERIALS AND METHODS**

**Construction, expression and purification of BLyS-gelonin.** Gelonin cDNA (Genbank accession# L12243) coding for amino acids G47-K297 was fused to the 5’ end of human BLyS cDNA (Genbank accession# NM_006573) coding for the secreted portion of BLyS consisting of amino acids A134-L285. Sequences were joined with sequence coding for a GGGGS linker and codon optimized for expression in *E. coli*.  In addition, a 6x His tag was added at the N-terminus followed by an Enterokinase site (DDDDK) to allow for the removal of the tag.  Sequences were cloned into an in-house *E. coli* expression vector using standard techniques. BLyS-gel was expressed in the cytoplasm of *E. coli* (Origami2, DE3 host; Novagen, Darmstadt, Germany) using an IPTG induction system. Origami2 cells containing BLyS-gelonin were cultured in 1 L shaker flasks in 2YT broth (Teknova, Hollister CA), then grown in 10 L bioreactors for approximately 26 hrs. The culture was induced with IPTG for 8 hrs. Cells were spun down and placed into plastic bags for short-term storage at -80°C (~600 g cell paste/10L culture). Frozen cells were thawed and reconstituted in lysis buffer (50 mM HEPES, 50 mM NaCl, and 2 mM MgCl_2_, pH 7; 30 ml buffer per 5 g cell paste) while stirring on ice. Complete, EDTA-free protease inhibitor cocktail minitablets (Roche, Indianapolis, IN) and Benzonase Nuclease (EMD Chemicals Inc., Darmstadt, Germany) were added to the bacterial solution. Reconstituted cells were subsequently lysed under pressure (7000 psi) using a microfluidizer apparatus to release BLyS-gel protein from the cytoplasm. The cell debris was spun down and the supernatant was loaded onto a nickel affinity column (GE, Uppsala, Sweden) in neutral conditions and eluted with 250 mM imidazole solution. Eluted samples were analyzed by western blot to identify the fractions containing BLyS-gel. These fractions were then pooled and subjected to a TACI affinity selection step in which active BLyS-gel was eluted with 0.1 M glycine HCl, pH 2.8. Fractions were neutralized with 1 M Tris then placed into dialysis cassettes (Pierce, Rockford, IL) for buffer exchange in dialysis buffer (20 mM sodium phosphate, 130 mM NaCl, pH 6.5). Endotoxin was removed with Cellufine resin (Chisso Corporation, Japan). The purity of BLyS-gel was evaluated by SDS-PAGE using NuPAGE Bis-Tris gels (Invitrogen, San Diego, CA) stained with Gelcode Blue (Pierce). The identity of purified BLyS-gel was confirmed by western blot analysis using antibodies specific for BLyS or gelonin.

**Determining the affinity of BLyS-gel for BLyS-receptors using surface plasmon resonance.**

Affinity analysis was performed using BIAcore 2000 and 3000 instruments. Soluble Protein A or Protein A/G was covalently immobilized on CM5 biosensor chips. The BLyS antibody or BLyS receptor-Fc fusions were then injected over the Fc capture molecule-derivatized flow cells at 30 μl/min for 10-15 sec. After washing off unbound ligand for 2.5 min with HBS-EP buffer (10 mM HEPES, pH 7.4, 150 mM NaCl, 3.4 mM EDTA, 0.005% Surfactant P20), a ligand density of between 150-250 RU was typically achieved. Serial dilutions of analyte (BLyS or BLyS-gel) were flowed at 15 μl/min for a total time of 1.67 min. The off rate of bound analyte was determined by washing the analyte-ligand-Protein A/G-complex in the presence of HBS-EP buffer for a period of 2.9 min. After each cycle, the flow cell surfaces were regenerated by washing twice with 10 μl of 10 mM glycine-HCl, pH 1.5. The regeneration cycle does not denature the derivatized surface, leaving it intact for subsequent injections. The procedure entailed flowing 8 two-fold serial dilutions of analyte (ranging from 0.078-10 μg/ml) and a single HBS-EP buffer injection, which constitutes a sample set. Each sample set was repeated 3 times. All binding analyses were performed at a constant temperature of 25°C. Data are reported as mean +/- 95% CI.

**Cell viability assay with primary B-CLL cells.** Frozen CD19^+^ cells isolated from the blood of B-CLL patients (Allcells, Emeryville, CA) were thawed, washed in RPMI + 10% FBS, and stained with trypan blue. Viability for all samples was >80%. Cells were then seeded in triplicate in opaque white 96-well polystyrene ½ area plates (Corning, Acton, MA) at a density of 5000 cells/well in 50 μl of RPMI + 10% FBS. An additional 50 μl of media was added containing titrations of BLyS-gel, gelonin, or bortezomib (Millennium). Cells were incubated at 37°C for 72 hrs and viability was measured by adding 40 μl of Cell Titer-Glo reagent (Promega) directly to cells in culture media. The cells were agitated for 5 min at room temperature and the luminescent signal was read using a Wallac Envision 2100 plate reader (Perkin-Elmer, Boston, MA).

**Internalization assay using primary B-CLL cells.** Cells were thawed as described above, washed in PBS, resuspended in FACS buffer, and incubated on ice with or without BLyS-gel (0.15 μg/100 μl cells) for 30 min. Cells were washed with ice cold PBS, then incubated another 0, 30, 60, or 120 minutes at 37°C. At each interval, cells were removed from 37°C and stained with rabbit anti-gelonin followed by a goat anti-rabbit PE (Southern Biotech). Propidium iodide (BD Pharmingen) was added for viability gating.
