# Supplementary figures and images for: Fusion Toxin BLyS-Gelonin Inhibits Growth of Malignant Human B Cell Lines In Vitro and In Vivo
Source: PLoS One. 2012 Oct 9;7(10):e47361. doi: 10.1371/journal.pone.0047361 (PMC3467252; doi:10.1371/journal.pone.0047361)

**A**

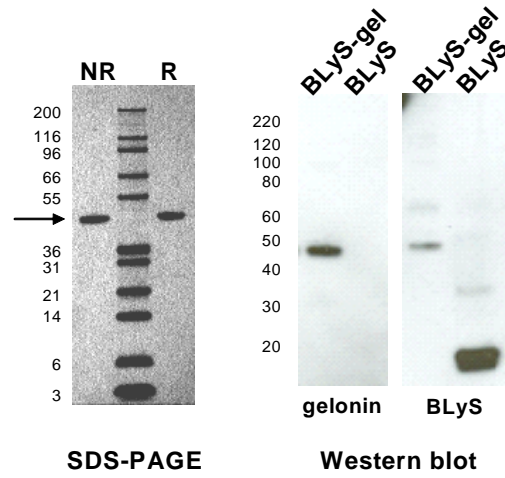

**B**

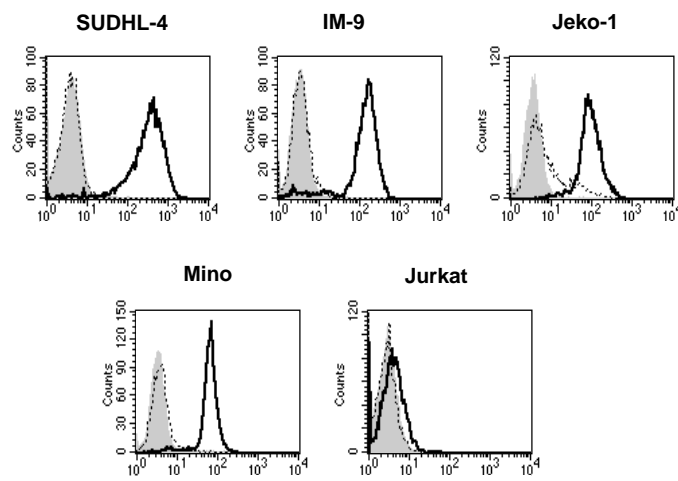

**C**

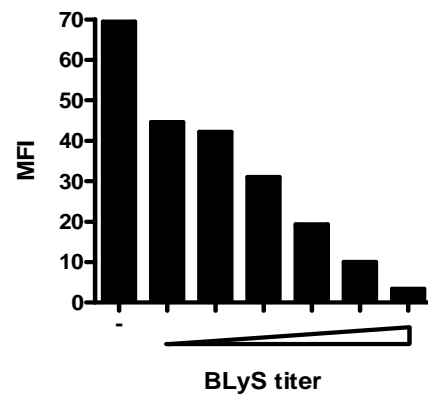

Supplement: Figure S1 — Characterization of BLyS-gel. A. Purified BLyS-gel was analyzed by SDS-PAGE under reducing (R) and non-reducing (NR) conditions. Gelcode Blue staining revealed a single band of approximately 45 kD (left panel), consistent with the expected size of BLyS-gel. Purified BLyS-gel or recombinant human BLyS were analyzed by western blot with anti-gelonin or anti-BLyS antibodies (right panels). Molecular weights (kD) are indicated to the left of each panel. B. Flow cytometric analysis of BLyS-gel binding to B & T cell lines. B cell lines SUDHL-4, IM-9, Jeko-1 and Mino all express at least one BLyS receptor (Fig. 1A and Table 1). The Jurkat T cell line, which does not express BLyS receptors, was used a negative control. Gray-shaded peak, gelonin detection reagents only; dashed line, gelonin+detection reagents; solid line, BLyS-gel+detection reagents. C. BLyS competes for binding of BLyS-gel to B cells. SUDHL-4 cells were incubated with 20 µg/ml BLyS-gel along with a titer of recombinant human BLyS from 80 ng/ml to 20 µg/ml. BLyS-gel binding was then analyzed by flow cytometry using an anti-gelonin antibody. Data are presented as the mean fluorescence intensity (MFI) of anti-gelonin stained cells. (PDF) [file pone.0047361.s002.pdf]

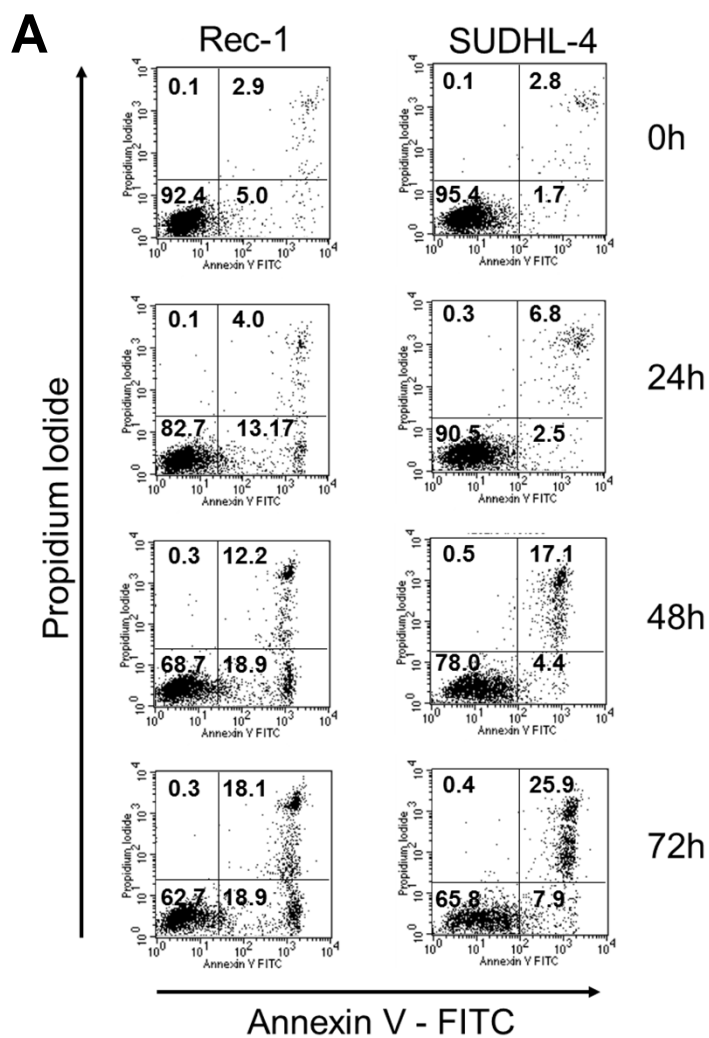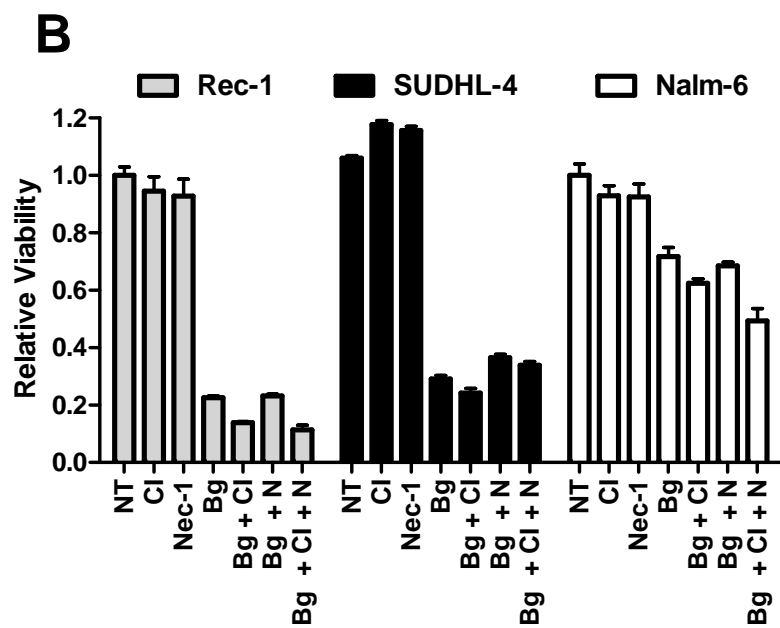

Supplement: Figure S2 — BLyS-gel treatment induces markers of apoptotic and necrotic cell death, but cytotoxicity is not blocked by caspase or necroptosis inhibitors. A. Rec-1 or SUDHL-4 cells were treated with BLyS-gel at 500 pM 0, 24, 48, or 72 hrs. Cells were then stained for phosphatidylserine exposure using annexin V and for membrane integrity using propidium iodide and analyzed by flow cytometry. The percentage of cells AxV−/PI− (viable), AxV+/PI+ (necrotic/dead) and AxV+/PI− (apoptotic) is shown in each quadrant. B. Rec-1 or Nalm-6 cells were treated with the indicated combinations BLyS-gel (Bg) at 500 pM, the general caspase inhibitor z-VAD-FMK (CI) at 10 µM, or the necroptosis inhibitor necrostatin-1 (N) at 10 µM. Cell viability was analyzed following 72 hrs of treatment. Data are presented as viability relative to untreated cells. NT, no treatment. (PDF) [file pone.0047361.s003.pdf]

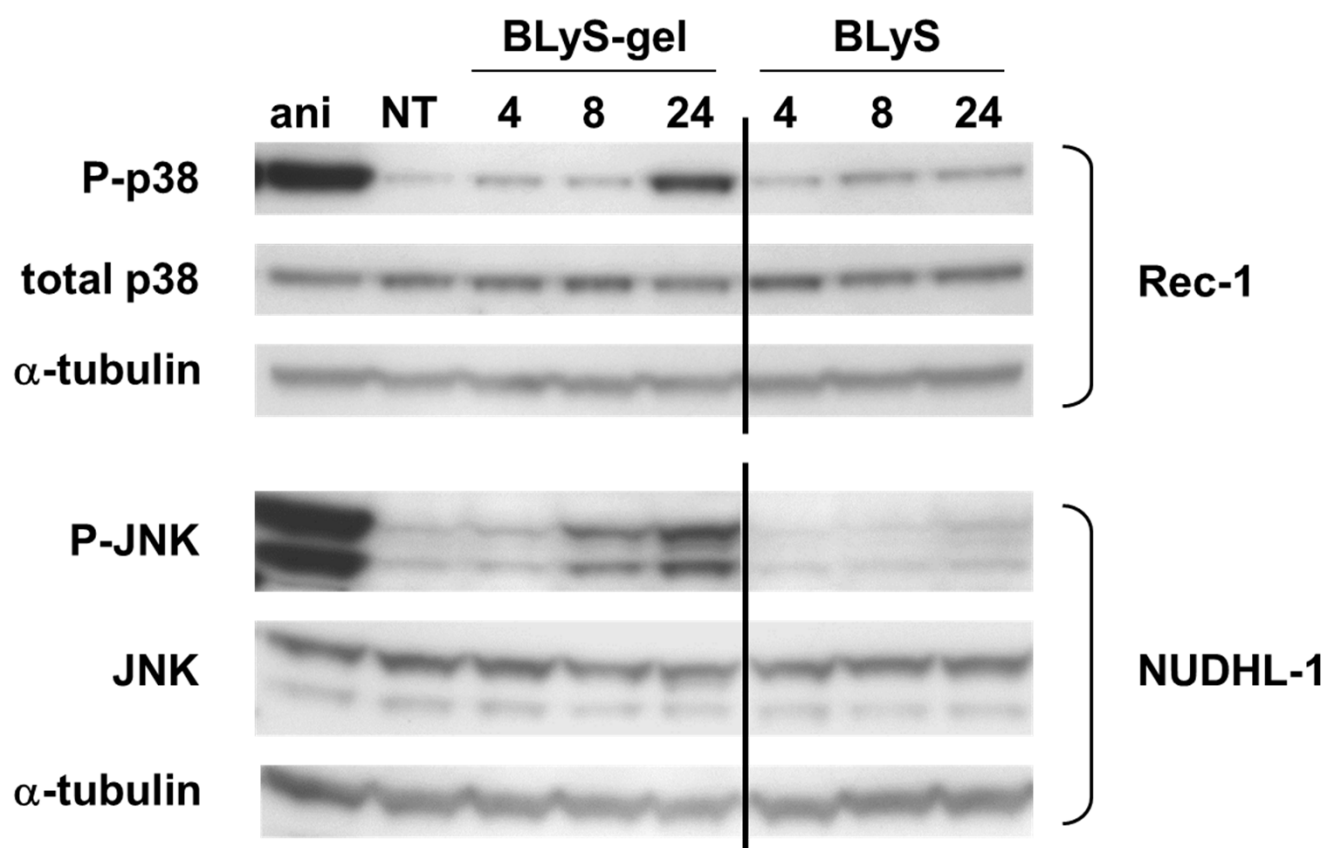

Supplement: Figure S3 — BLyS-gel, but not BLyS, induces p38 and JNK phosphorylation. Rec-1 or NUDHL-1 cells were treated with BLyS-gel or BLyS at 500 pM for 4, 8 or 24 hrs prior to collection of whole cell lysates for western blot analysis. Cells were also treated with anisomycin (ani) as a positive control for induction of p38 and JNK phosphorylation. Blots were probed using antibodies specific for phosphorylated and non-phosphorylated forms of p38 or JNK. α-tubulin was probed as a loading control. (PDF) [file pone.0047361.s004.pdf]

**A**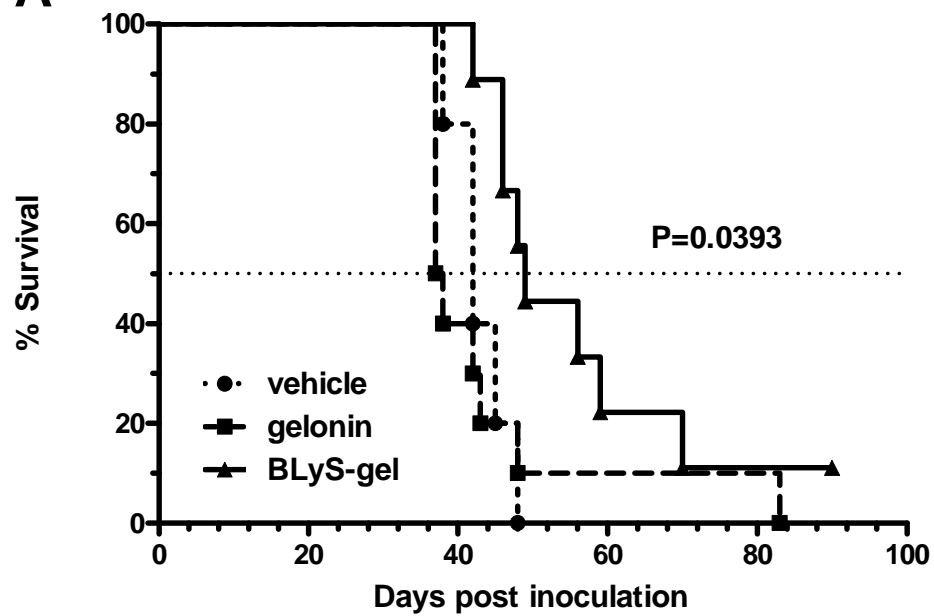**B**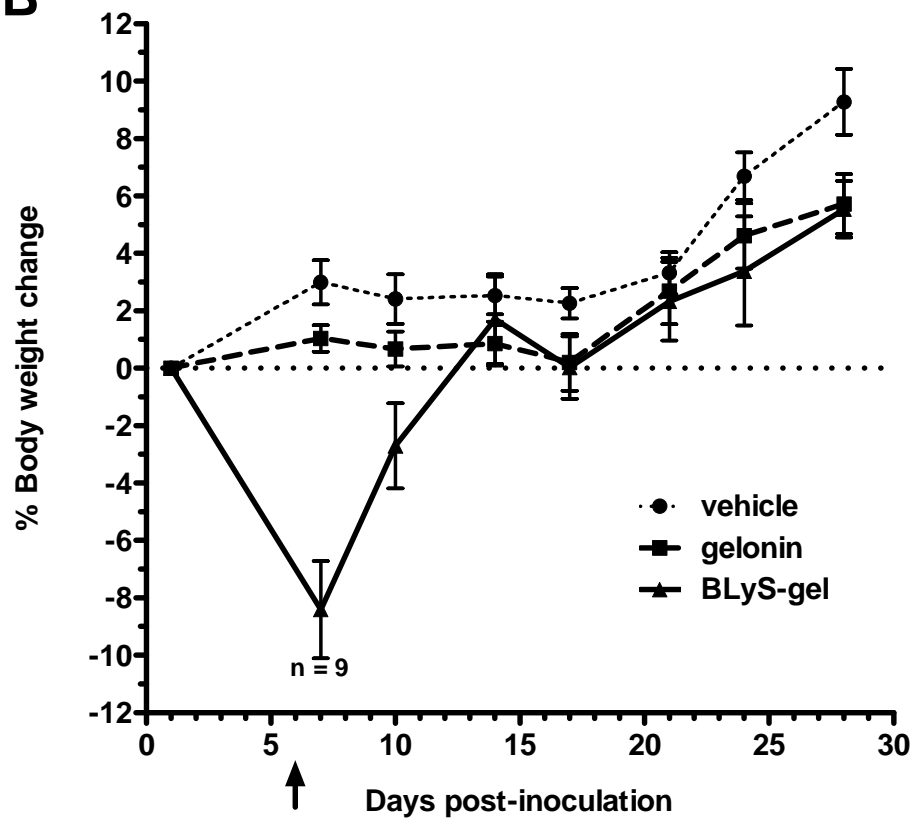

Supplement: Figure S4 — BLyS-gel treatment effects on survival and body weight in the Nalm-6 model of BCP-ALL. Nalm-6 BCP-ALL cells (1×10^6^) were injected into the tail veins of SCID mice on day 0. Mice were divided into three groups (n = 10) for i.v. treatment with free gelonin, BLyS-gel, or an equivalent volume of vehicle. All mice were injected with the murine BLyS-specific antibody 10F4 (5 mg/kg) to deplete circulating mBLyS on day 1, and treatments began on day 2. Mice were treated (2 mg/kg) on days 2–6. A. Kaplan-Meyer survival curve. P value refers to results of the Logrank test. B. Percent body weight change. On day 6, one of the BLyS-gel treated animals died as result of apparent treatment related toxicity, reducing the number of mice in this group to 9. Arrow indicates the last day of treatment. (PDF) [file pone.0047361.s005.pdf]

**A**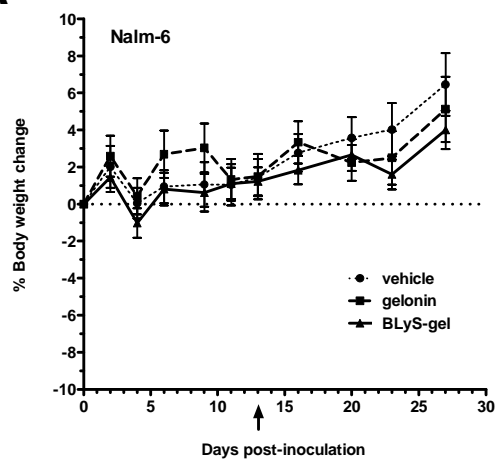**B**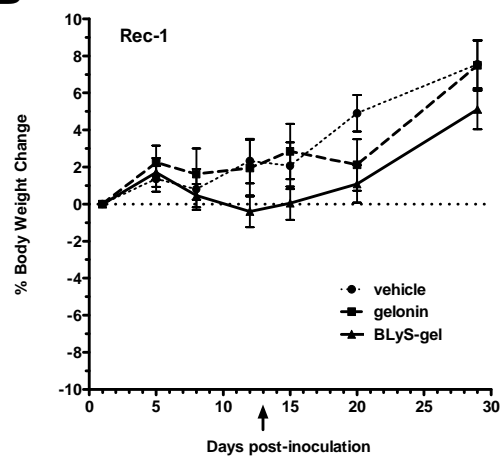**C**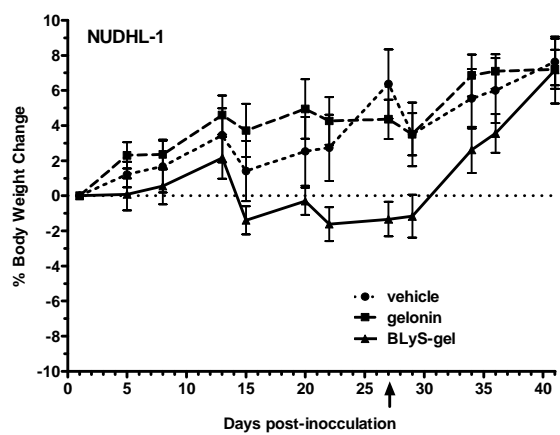**D**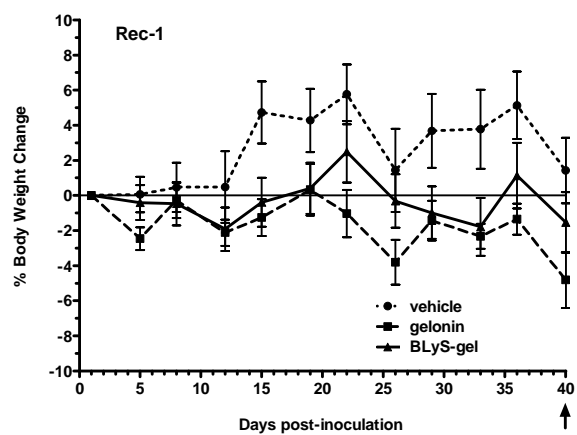

Supplement: Figure S5 — BLyS-gel treatment effects on body weight in various models. Nalm-6 BCP-ALL, Rec-1 MCL, or NUDHL-1 DLBCL cells (1×10^6^) were injected into the tail veins of immunodeficient mice on day 0. Mice were divided into three groups (n = 10) for i.v. treatment with free gelonin, BLyS-gel, or an equivalent volume of vehicle. All mice were injected with the murine BLyS-specific antibody 10F4 (5 mg/kg) to deplete circulating mBLyS on day 1, and treatments began on day 2 in all studies. Arrows indicate the last day of treatment. A. Mice were treated (2 mg/kg) 3 times per week for 2 weeks. B. Mice were treated (2 mg/kg) 3 times per week for 2 weeks. C. Mice were treated (1 mg/kg) twice per week for 4 weeks. D. Mice were treated (2 mg/kg) twice per week for 6 weeks. (PDF) [file pone.0047361.s006.pdf]

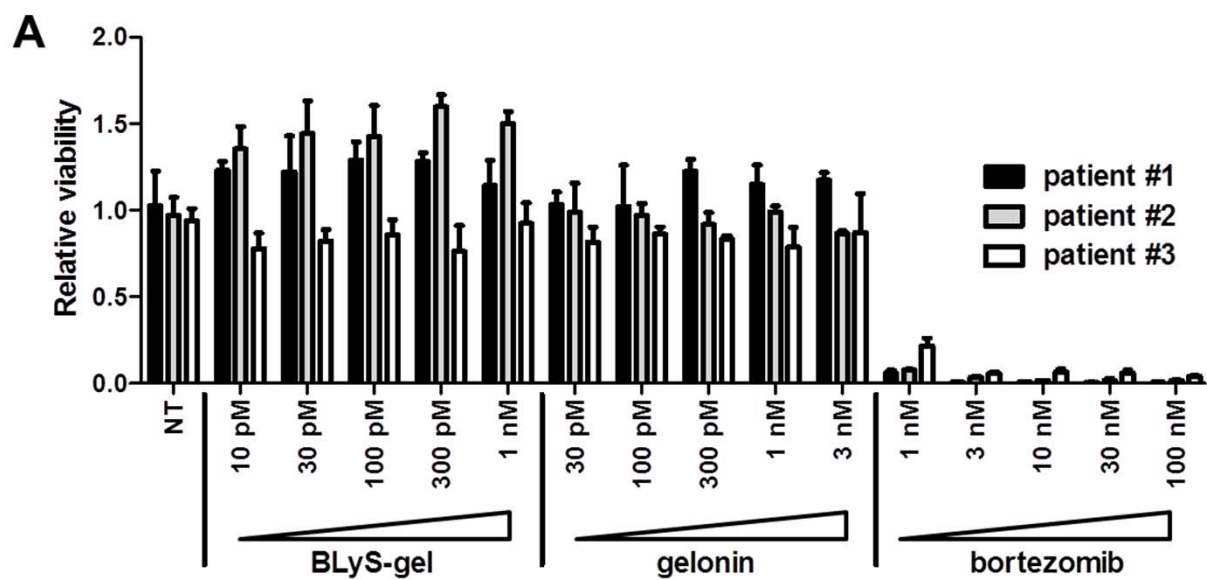

**B**

Cell surface expression of BCMA, TACI, and BR3

| Sample     | BCMA | TACI | BR3  | BLYS |
|------------|------|------|------|------|
| patient #1 | 0.4  | 1.8  | 52.3 | 66.8 |
| patient #2 | 0.9  | 2.5  | 36.1 | 28.1 |
| patient #3 | 5.4  | 15.6 | 96.8 | 70.7 |

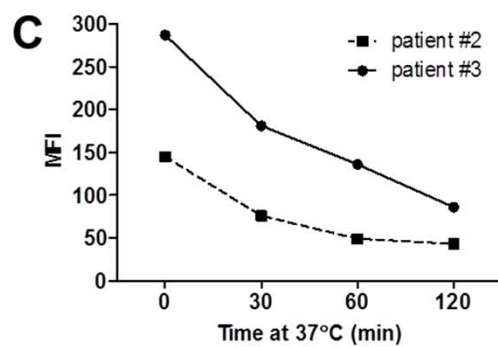

Supplement: Figure S6 — BLyS-gel effects on primary B-CLL cells. A. CD19+ cells isolated from the blood of three B-CLL patients were thawed and treated with a titer of BLyS-gel, gelonin, or bortezomib. Cell viability was analyzed following 72 hrs of treatment. Data are presented as viability relative to untreated cells. NT, no treatment. B. Primary B-CLL cells were analyzed for BLyS receptor expression by flow cytometry. Samples were stained with antibodies specific for BCMA, TACI or BR3. Alternatively, the ability of cells to bind BLyS was determined by incubation with biotinylated BLyS. Values shown represent the mean fluorescence intensity (MFI). C. BLyS-gel internalization into primary B-CLL cells was analyzed by flow cytometry. Samples were incubated with BLyS-gel for 30 min at 4°C, then transferred to 37°C for 30 to 120 min to allow BLyS-gel to internalize. The remaining surface bound BLyS-gel was then detected using a gelonin-specific antibody. The decrease in MFI over time at 37°C indicates internalization has occurred. (PDF) [file pone.0047361.s007.pdf]
