# Supplementary material for: Fusion Toxin BLyS-Gelonin Inhibits Growth of Malignant Human B Cell Lines In Vitro and In Vivo
Source: PLoS One. 2012 Oct 9;7(10):e47361. doi: 10.1371/journal.pone.0047361 (PMC3467252; doi:10.1371/journal.pone.0047361)
Supplement: Table S1 — BLyS-gel binds BLyS-receptors. Affinity data for BLyS-gel binding to BLyS-receptors determined by surface plasmon resonance. (PDF) [file pone.0047361.s008.pdf]

Supplementary Table 1. BLyS-gel binds BLyS-receptors

Receptor	Ligand	Binding Affinity
anti-BLyS mAb	BLyS-gel	k_d = 324 +/- 107 pM
anti-BLyS mAb	BLyS	k_d = 250 +/- 119 pM
TACI-Fc	BLyS-gel	k_d = 0.8 +/- 0.5 nM
TACI-Fc	BLyS	k_d = 1.6 +/- 0.2 nM
BAFFR-Fc	BLyS-gel	k_d = 2.6 +/- 1.4 nM
BAFFR-Fc	BLyS	k_d = 2.2 +/- 0.7 nM
BCMA-Fc	BLyS-gel	k_d = 6.2 +/- 2.5 nM
BCMA-Fc	BLyS	ND

* mean +/- 95% CI, n=3
