# Supplementary material for: Fusion Toxin BLyS-Gelonin Inhibits Growth of Malignant Human B Cell Lines In Vitro and In Vivo
Source: PLoS One. 2012 Oct 9;7(10):e47361. doi: 10.1371/journal.pone.0047361 (PMC3467252; doi:10.1371/journal.pone.0047361)
Supplement: Table S2 — mBLyS concentration in blood of SCID mice. Quantification of murine BLyS levels before and after injection of the mBLyS-specific antibody 10F4. (PDF) [file pone.0047361.s009.pdf]

Supplementary Table 2. mBLyS concentration in blood of SCID mice (ng/ml)*

Sample	Days following i.v. injection of 10F4 (5 mg/kg)					
	0h	1d	2d	3d	4d	5d
1	69.2	2.1	-	-	-	-
2	74.2	BLOQ	-	-	-	-
3	85.8	-	2.9	-	-	-
4	74.5	-	8.6	-	-	-
5	81.9	-	-	2.1	-	-
6	87.9	-	-	2.9	-	-
7	90.8	-	-	-	BLOQ	-
8	79.4	-	-	-	3.8	-
9	95.5	-	-	-	-	6.2
10	91.4	-	-	-	-	BLOQ

*values determined by quantitative ELISA using mBLyS standard curve
Abbreviations: BLOQ, below limit of quantitation (0.8 ng/ml)
