# Supplementary material for: Fusion Toxin BLyS-Gelonin Inhibits Growth of Malignant Human B Cell Lines In Vitro and In Vivo
Source: PLoS One. 2012 Oct 9;7(10):e47361. doi: 10.1371/journal.pone.0047361 (PMC3467252; doi:10.1371/journal.pone.0047361)
Supplement: Table S3 — hβ2M concentration in mouse serum. Quantification of human β2M levels in serum of mice inoculated with Rec-1 cells. (PDF) [file pone.0047361.s010.pdf]

Supplementary Table 3. hβ2M concentration (ng/ml) in mouse serum*

Mouse	Week 1	Week 2	Week 3	Week 4	Week 5	Week 6
1	BLOQ	BLOQ	BLOQ	6.90	8.20	8.39
2	BLOQ	BLOQ	BLOQ	2.58	7.87	8.72
3	BLOQ	BLOQ	BLOQ	3.26	8.83	8.27
4	BLOQ	BLOQ	BLOQ	5.66	8.68	8.78

*mice were injected i.v. with $1x10^6$ Rec-1 cells. Serum was collected each week and analyzed for the presence of human β2-microglobulin (hβ2M) by ELISA as described in Materials and Methods.
